# Comprehensive High-Spatial-Resolution Imaging Metabolomics Workflow for Heterogeneous Tissues

**DOI:** 10.1021/acs.analchem.4c05410

**Published:** 2025-05-12

**Authors:** Xin Diao, Jianing Wang, Chengyi Xie, Leijian Chen, Thomas Ka-Yam Lam, Lin Zhu, Zongwei Cai

**Affiliations:** † State Key Laboratory of Environmental and Biological Analysis, 26679 Hong Kong Baptist University, Kowloon, Hong Kong SAR 999077, China; ‡ Department of Chemistry, Hong Kong Baptist University, Kowloon, Hong Kong SAR 999077, China; § School of Marine Science and Engineering, Hainan University, Haikou 570228, China; ∥ Eastern Institute of Technology, Ningbo, Zhejiang 315200, China

## Abstract

Mass spectrometry imaging is a developing technique that maps the molecular composition of samples in a label-free manner. However, highly heterogeneous samples, including bones, usually cannot be easily analyzed due to challenging sample preparation, particularly in minimizing cracks and maintaining flatness. To comprehensively address these issues, we developed a sample preparation method for the fresh frozen heterogeneous samples such as knee joint and skull of murine, which includes complex structures and tissue types, such as neuronal tissue, peripheral nerve, muscle, tracks, connective tissue, cartilage, mineralized bone, and bone marrow. By controlling the sample thickness and employing an optimized drying method, we achieved minimal cracking. We found that a combination of lyophilization and 5 μm section thickness, when attached to a cryofilm, was readily achievable and significantly reduced cracking in bone tissue. Additionally, we implemented a contactless spin-flattening technique to ensure surface uniformity. Centrifuging the section at 7000*g* improved surface flatness, bringing the height variation within the range typically observed in soft tissues while also removing excess mounting medium and bubbles. This approach enhances the sample quality and reliability without requiring complex manual skills, making it more practical and reproducible for routine analysis. High molecular coverage was achieved, including small metabolites, metals, and lipids, by using the *N*-(1-naphthyl) ethylenediamine dihydrochloride (NEDC) matrix. To further explore the potential of our workflow, high-resolution MSI was performed on rat tibial growth plates at different growth stages. Numbers of *N*-acetyl disaccharide sulfate and PE (34:1) are found to be complementary expressed in growth plate cartilage and have different intensities at different growth stages. Our findings suggested the potential involvement of those metabolites in bone development. By addressing the challenges of sample preparation, including surface flatness, bubbles, and severe cracking, our approach significantly improves the quality of the MS imaging. Additionally, this method offers a broad detection range that encompasses both metal ions and metabolites. This advancement enables detailed and accurate molecular characterization of rigid biological samples, enhancing the potential for applications in biomedical research.

## Graphical abstract

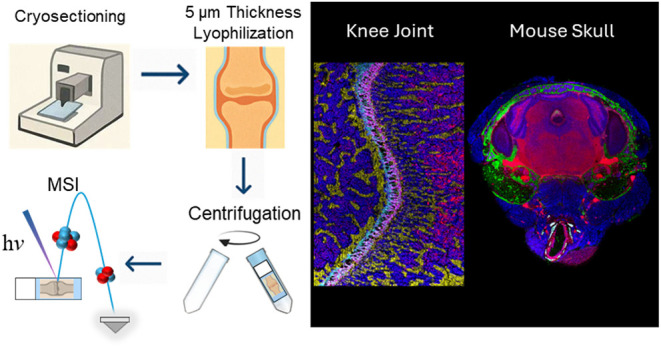

## Introduction

Metabolite imaging is crucial for studying in situ metabolite distribution, but it presents significant challenges. Optical microscopy heavily relies on immunological labeling and target-specific fluorophores. Both limitations in antibodies and overlapping fluorophore emission spectrum prevent the depth of optical imaging. The development of multiplexed immunoaffinity techniques, such as co-detection by indexing (CODEX),^1^ cyclic immunofluorescence microscopy,^2,3^ and multiplexed ion beam imaging (MIBI),^4^ enables dozens of targets to be imaged in one experiment. However, in the regime of the metabolome, in situ detection is much more challenging due to the lack of target-specific tags when using labeling techniques. Other label-free techniques like nuclear magnetic resonance imaging (MRI) in combination with magnetic resonance spectroscopy (MRS) could spatially quantify several metabolites, such as choline, lactate, and inositol.^5^ While these methods provide valuable information for both clinical investigation and research studies, they are limited by their low spatial resolution, coverage, and sensitivity.

Mass spectrometry imaging is a developing technology that spatially resolves molecular composition in a label-free manner.^6–8^ MALDI-basedmass spectrometry imaging has been widely implemented from suborgan spatial metabolomics discoveries to single-cell metabolomics dynamics, with increasing spatial resolution.^9–11^ Nevertheless, some pending challenges in mass spectrometry imaging limit the application of mass spectrometry imaging in spatially resolved metabolomics, hindering its broader use in more heterogeneous biological samples such as bone and skull. Generally, MALDI MSI prefers fresh-frozen samples that preserve morphology and are valuable in situ information on metabolites. Highly heterogeneous tissues, such as the joints and skull, containing soft tissue and mineralized structure, as well as varying densities and mechanical properties, make it difficult to obtain high-quality sections that meet current research requirements. Developing an adhesive film-based section method (Kawamoto’s method) provides structural support and helps transfer the tissue slice after sectioning, making imaging of fresh frozen bone achievable.^12^ Several reported research successfully incorporated Kawamoto’s method into the conventional MALDI MSI workflow.^13–17^ However, technical difficulties remain to be solved, including postsection cracks, surface flatness, and metabolome coverage. Recent studies on fresh-frozen sections of mouse tibia combined freeze-drying and sublimation to achieve 10 μm resolution imaging.^14^ Although this work significantly improved in containing cracks and artifacts compared to previous work, even at high spatial resolution,^16–18^ matrix sublimation generally resulted in reduced ion intensities and prevented high-coverage imaging metabolomics.^19–21^ The crack issue remains problematic at high spatial resolution because the relative impact of cracks is still significant.^14–18^ Consequently, further investigation is needed to develop spray-based matrix deposition and sample preparation methods that better control the incidence of cracks and improve the molecular coverage.

Surface flatness is crucial for MALDI MSI since defocus laser beams cannot efficiently desorb and ionize analytes from the sample surface.^22^ Irregularities in surface topography can lead to uneven laser focus, resulting in poor desorption and ionization efficiency, and affecting the spatial resolution and sensitivity of the imaging. Schaepe et al. reported imaging of metabolites in native human bone using three different ionization techniques including scanning microprobe matrix-assisted laser desorption/ionization.^22^ They developed a metal supporting frame to support the cryofilm. The metal frame was glued to the top of the cryofilm to stretch the cryofilm. However, the off-focus problem still remains to be solved. Bender et al. developed a sample preparation workflow for mass spectrometry imaging of fresh frozen spines of mice.^15^ A polytetrafluoroethylene roller-based film mounting strategy was introduced to maintain an even surface of the sample. Even though PTFE roller-based flattening could obtain a relatively smooth surface, it also raised the concern that direct contact with the PTEF roller with tissue might cause delocalization of nonpolar metabolites.^23^ Additionally, this method might struggle to apply uniform pressure, particularly on highly stressed, curled tissues. Thus, batch effects and reproducibility might not be satisfactory. Khodjaniyazova et. al also reported a strategy that bypasses the need for cryosections to image fresh frozen bone.^24^ This method is tailored for IR-MALDESI techniques. The fresh bone was cut in half and positioned with a flat surface downward in the mold. After being embedded in the Plaster of Paris, the flat surface can be directly imaged with an IR-MALDESI mass spectrometer. However, the inherent problem of nonsectioned samples might prevent its application in commercial MALDI spectrometers. Therefore, a universal contactless cryofilm flattening method is still in demand and needs to be developed to improve the quality of the MSI of highly heterogeneous tissue.

Metabolome coverage or imaging depth is another consideration in MSI since it is directly related to deciphering molecular information, especially in pathological contexts. Rigid biological samples usually contain several tissue and cellular types, which makes them also highly heterogeneous in the metabolome. Metabolites play a crucial role in maintaining normal physiological function and metabolic balance of tissue.^25,26^ Comprehensively detecting endogenous molecules assisted in the better understanding of the causes and progression of the disease. Endogenous calcium, phosphorus, vitamin D, and chondroitin are essential for the growth, development, and maintenance of rigid tissue such as bones.^27,28^ Thus, a comprehensive molecular imaging method with both inorganic and organic coverage is demanded and important. A comprehensive rigid sample MSI workflow with a high molecular coverage is still lacking.

In this study, we reported an optimized workflow for mass spectrometry imaging of highly heterogeneous fresh-frozen tissue, which comprehensively addresses the challenges associated with such complex samples. By evaluating postsection cracks by different section thicknesses and drying methods, we found that a combination of thinner sections and lyophilization-drying generated minimal cracks supported up to 10 μm spatial resolution mass spectrometry imaging without significant artifacts and signal loss. We developed a novel sample mounting method, spin-flattening, using high-speed centrifugation, which ensures exceptionally flat sample mounting by effectively eliminating any curling and bubbles. This innovative approach supports large-area specimens, including entire mouse skulls, and is not limited to small objects, expanding its applicability and ensuring high-quality imaging. *N*-(1-naphthyl) ethylenediamine dihydrochloride (NEDC) was chosen as the MALDI matrix for its excellent coverage for lipids and low background at low *m*/*z* range, which benefits both lipids and small metabolites detection.^9,10,29–31^ More importantly, the reported study also addressed the ability of NEDC to detect endogenous metal levels,^32^ which is crucial for highly heterogeneous tissue imaging. We benchmarked it with rodents’ skull and knee joints and comprehensively studied its molecular composition by MALDI MSI. Abundant endogenous molecules were detected, including inorganics, amino acids, nucleosides, glycerophospholipids, glycosaminoglycans, and free fatty acids. Notably, by using NEDC as a negative-ion mode matrix, the spatial distribution of seven metal ions was also determined, highlighting the superior imaging depth of our workflow. Several metabolites were found to be spatially regulated and might be associated with bone development. Further high spatial resolution imaging was performed to investigate molecular differences between rat bone cartilage at different ages.

## Experimental Section

Chemicals and materials and additional experimental details are provided in the Supporting Information.

### Sample Preparation

Embedding medium containing 10% gelatin and 5% carboxymethyl cellulose was dissolved in water and heated until it turned clear. The embedding medium were kept at 37 °C to prevent gelation. Once the knee joint and skull of the rats and mouse were isolated, it was immediately frozen in liquid nitrogen with embedding medium and stored at −80 °C. Before cryosectioning, tissue blocks were equilibrated to −20 °C. After 30 min of temperature equilibration, the tissue block was trimmed on a CryoStar microtome until an interesting cross-section was observed. A cryofilm was directly applied to the bone tissue and sectioned to a 5 μm thickness. The tissue section was transferred into a lyophilizer using a prechilled container maintained at −20 °C and dried in the lyophilizer for 2 h. Then, the tissue section was mounted onto anindium tin oxide glass slidewith ZIG 2-way glue with one end mounted with clean room tape (VWR International). Then, the slides were placed in a 50 mL conical centrifuge tube filled with cotton and spun in an angular rotor at 7000*g* for 10 min to remove excess adhesive and flatten the surface.

### Matrix Deposition and MS Imaging

For the positive mode, 13 layers of dihydroxybenzoic acid (20 mg/mL in 70% methanol) were directly deposited by a home-built pneumatic sprayer. Sprayer nozzles were heated to 75 °C, and the sprayer velocity was set to 1920 mm/min with CC motion. Matrix solution is nebulized with 10 psi of nitrogen flow with a 100 μL flow rate. Ten layers of *N*-(1-naphthyl) ethylenediamine dihydrochloride prepared in 70% methanol (5 mg/mL) were deposited at a 10 μL/min flow rate. Mass spectrometry imaging was conducted on a timsTOF flex MALDI 2 instrument (Bruker Daltonics). For 30 μm lateral resolution imaging, the laser was operated at 10,000 Hz with 200 accumulated laser shots. For 10 μm lateral resolution imaging, the laser was operated at 5000 Hz with 100 accumulated shots per pixel. The detection range was set to cover the *m*/*z* 50–1500 range.

### Metabolites Identification and Assignment

Before imaging experiments, spot mass spectra were obtained at different structures of the tissue section. Then, mass spectra were imported into the DataAnalysis (Bruker Daltonics), and the mass peaks list was generated at a threshold of signal-to-noise ratio (S/N) greater than 3 with the centroid processing mode. The peaks list was searched against the Human Metabolome Database (HMDB) (https://hmdb.ca/) and Lipid Maps database (https://www.lipidmaps.org/) with five ppm mass tolerance. For the positive-ion mode, protonation, sodium, and potassium adduction were enabled, while deprotonation, chloride adduction, and demethylation were enabled in negative-ion mode. Ions of interest were further identified with direct infusion MS of the tissue extract. Bone sections were collected during cryosection. After the region of interest was carefully dissected with a blade, it was placed in a 1.5 mL Eppendorf tube with forceps. Then, 500 μL of 80% methanol was added and vortexed at high speed for 2 min. Tissue homogenate was then centrifuged at 20,000*g* for 15 min, and the pellet was discarded. The supernatant was then concentrated in a SpeedVac vacuum concentrator. The tissue extract was first reconstituted in 100 μL of chloroform-methanol (1:1, v/v) and further diluted 100 times in methanol. The resulting solution was directly infused into an Orbitrap Fusion mass spectrometry with a resolution of 120,000 at 200 *m*/*z*. The tandem mass spectrometry setting of individual ions is summarized in Supporting Table S3.

### Data Processing

For the MSI experiment, raw data files were imported and processed by the SCiLS Lab (Bruker Daltonics) using the default setting. Segmentations were performed with an aligned peak list with built-in Move Peaks to local Maximum function. All spectra were root-mean-square normalized. *K* means clustering with eight classes was implemented for the mouse skull and rat joint.

## Results and Discussion

### Development Workflow for Highly Heterogeneous Samples with High Coverage

Several reported studies have successfully incorporated film-based cryosection with MALDI MSI.^13–18,33^ The addition of cryofilm supported the simple sectioning and also limited the performance of MS imaging of fresh frozen rigid samples. Tissue cracking is one of the major concerns, especially at high spatial resolution, which causes metabolite relocation and morphological artifacts. The extra cryofilm mounting step reduces the success rate of MALDI MSI due to uneven surface and laser defocus. Here, we provided an optimized workflow for highly heterogeneous samples, from sample preparation to data acquisition, with contactless sample mounting and a high metabolome coverage feature (Figures 1 and S1). The adult mouse skull was concurrently snap-frozen with previously reported embedding medium.^34^ Kawamoto’s method was implemented to help with the sample sectioning, and an adhesive tape was attached to the surface of the embedded tissue to maintain the specimen’s morphology without fixation and decalcification. After cryosection, 5 μm thick sections were transferred into a vacuum chamber without thawing to reduce tissue cracking. Then, tissue sections were mounted onto an ITO-coated glass by centrifugation to achieve high flatness and minimal trapped air.

**Figure 1:**
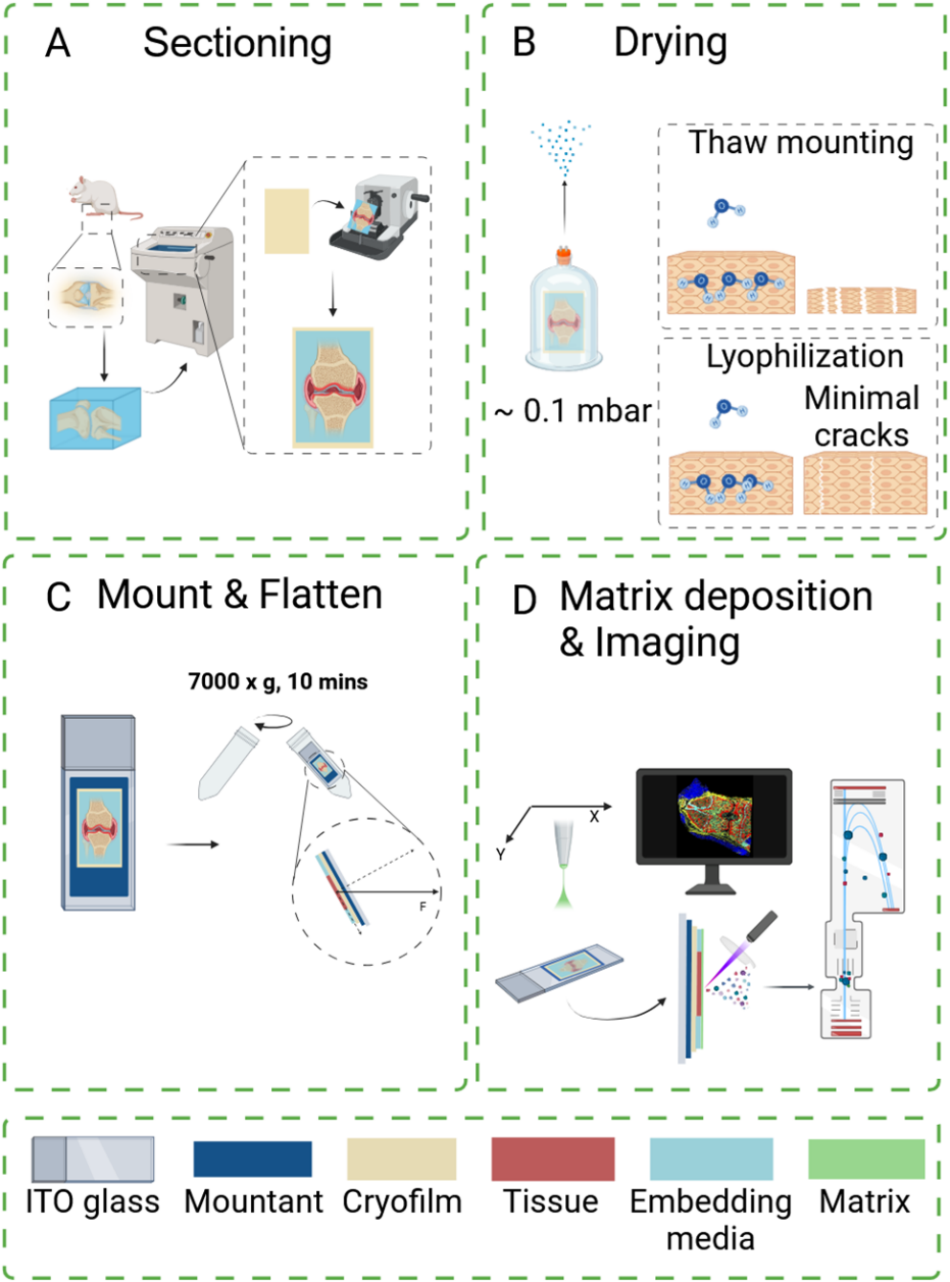
Schematic workflow of comprehensive high-spatial-resolution imaging metabolomics workflow for heterogeneoustissue. (A) Tissue was embedded in 10% gelatin/5% CMC and sectioned with the assistance of cryofilm. (B) The tissue section was transferred to lyophilization. (C) Dried tissue section was mounted and fattened by spin-flattening technique using centrifugation. (D) Matrix application and MALDI-MSI. *Created in BioRender. Wang, J. (2025)* https://BioRender.com/b78p083.

We first investigated the parameters that affect crack formation during drying. Due to the high heterogeneity in texture, cracks usually form during the thawing process. Reported literature suggested that a combination of lyophilization and matrix sublimation could greatly help with crack control.^14^ We believe that tissue thickness and drying methods are deterministic factors of postsection cracks. We measured the crack width of mouse skull sections of 5–14 μm thickness dried with freeze-drying and thawing mounting (Figure 2). In general, tissue drying by lyophilization results in smaller cracks (Figure 2A), and this is in line with reported literature.^14^ Different textures or molecular compositions have significant effects on postsection cracks, such as in the brain and muscles (Figure 2B). By reducing the thickness of tissue from 14 to 5 μm, postsection cracks reduce accordingly, regardless of the composition of tissue (Figure 2C,D). As section thickness increases, postsection cracks increase accordingly and obviously at bright-field scan (Figure S2A). At 14 μm section thickness, cracks significantly affect the imaging quality of 30 μm lateral resolution MALDI MSI, and no obvious cracks were observed at the 5 μm section (Figure S2B). In addition, the thinner section has no significant effect on the detection of metabolite signals at lower and higher mass regions (Figures 2B,C and S3). After drying, moisture evaporates from the tissue section, causing shrinkage. However, mineralized parts would not shrink as soft tissue (brain and muscles) creates tension in the tissue. Lyophilization directly sublimates moisture into the gas phase, which might explain the smaller cracks found on lyophilized sections. For mass spectrometry imaging of the entire mouse skull at 30 μm lateral resolution, the combination of lyophilization and 5 μm section thickness results in no obvious cracks in either bright-field scan or ion imaging (Figure S2A). Our results demonstrate that lyophilization significantly reduces tissue cracks, consistent with the findings of Good et al.^14^ Moreover, we found that reducing the section thickness to 5 μm further minimizes cracks, leading to improved section quality for high-resolution mass spectrometry imaging.

**Figure 2:**
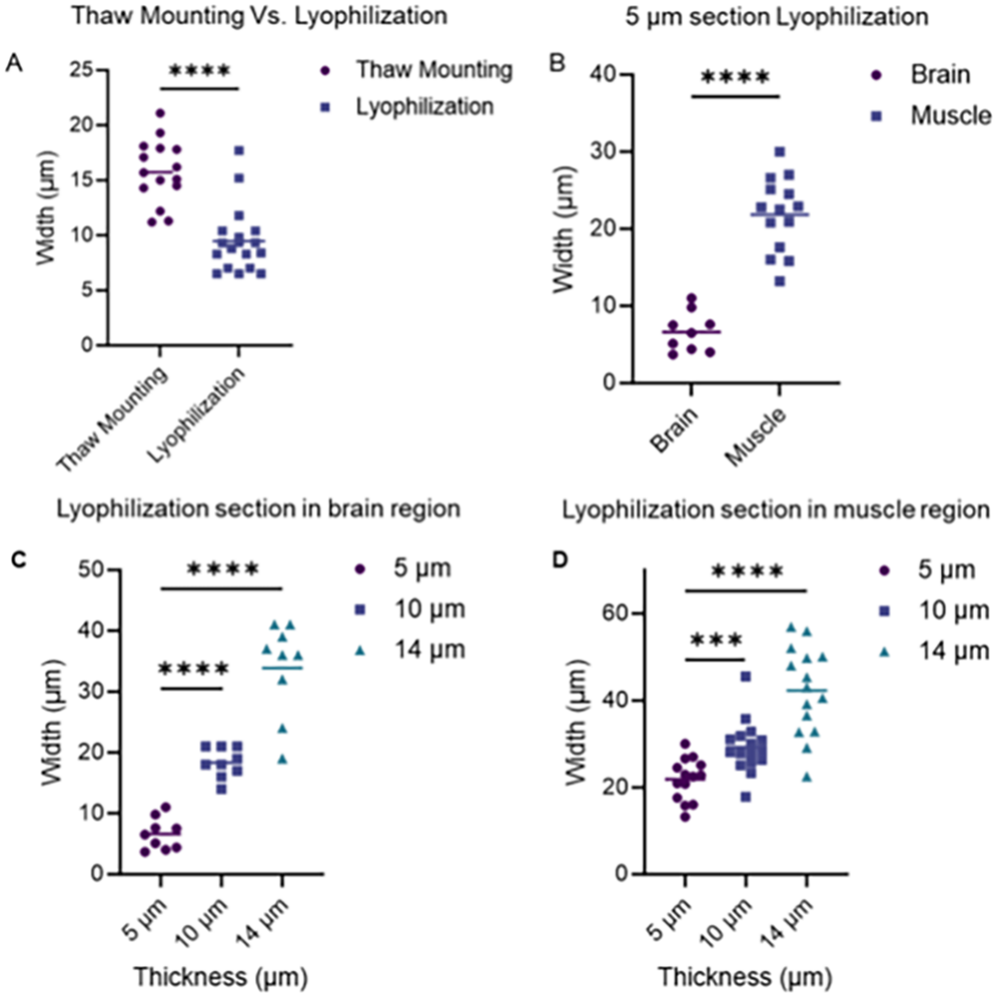
Crack width of the mouse skull section was prepared in different conditions. Cracks width generated on mouse brain dried by thaw mounting and lyophilization (A). Comparison of cracks in muscle and brain on 5 μm skull section using lyophilization (B). Crack width of different thicknesses of mouse skull in brain region after lyophilization (C). Crack width of different thicknesses of mouse skull in muscle after lyophilization (D).

High-quality MALDI MSI is ensured by minimizing the surface unevenness. Since the sample was attached to cryofilm by adhesive, an additional mounting step is required to transfer the section onto a glass slide. Previous methods of directly attaching adhesive tape to the ITO slide with ZIG 2-way glue or similar ways usually resulted in an uneven surface and trapped air bubbles. The uneven thickness of the mountant and trapped air bubbles cause misalignment of the laser focus, leading to oversampling, reduced signals, or no signals, which creates signal reduction or empty spots in ion images.^15,22^ To address this issue, we developed a contactless method to flatten the cryofilm onto surfaces with minimal bubbles and unevenness. Excess glue was used to mount the microfilm-tissue surface on the ITO slides to ensure that minimal bubbles were generated during the mounting. Then, the ITO slide with the mounted sample was loaded on an in-house slide rack and centrifuged at 7000*g* on an angular rotor (Figure S4). Excess glue was drained during centrifugation without specimen contamination, and cryofilm was flattened to the ITO slide surface. We measured the variation in the height of the fresh-frozen brain section, spin-flattened bone section, and direct-mounted bone section (Figure S5A). Compared with the thaw-mounted brain section, the spin-flattened bone section does not have higher unflatness (Figure S5B). The direct-mounted bone section has a much higher height due to the presence of air bubbles and nonuniform mountant. Compared with existing techniques dealing with nonflatten samples using roller and metal frames,^15,22^ the current methods have easy-to-adopt and contactless features. It is a comprehensive workflow designed to address the key challenges in MSI sample preparation, including postsection cracking, warping, and surface irregularities.

Molecular coverage is another key consideration for MALDI-MSI. Previously reported studies,^14–17,35^ regardless of decalcified or fresh frozen tissue, only have limited molecular coverage in small metabolites and lipids. *N*-(1-naphthyl) ethylenediamine hydrochloride is a widely adopted MALDI matrix for its known low signal interference at a low *m*/*z* range and high metabolite coverage for small polar metabolites and lipids.^9,10,29–31^ It has been noted that NEDC has reported excellent ability to detect metal ions.^32^ Considering the molecular complexity of heterogeneous tissues and the coverage of NEDC in inorganic and organic molecules, it is reasonable to choose NEDC as a matrix to detect the spatial molecular distribution of heterogeneous tissues. To test whether NEDC is suitable for rigid sample MALDI-MSI, skull sections of mice were deposited with NEDC through an in-house-built automatic electrospray platform. Then, mass spectra were acquired with a 30 μm laser ablation spot in negative-ion modes in a conventional axial MALDI time-of-flight (TOF) instrument (Rapiflex, Bruker Daltonics). Positive mass spectra were acquired on the same instrument with 2,5-dihydroxyl benzoic acid. Regardless of the ionization mode, abundant metabolite and lipid signals were detected (Figures S6A and S7). To further evaluate the coverage of our method, the sample was measured in a higher mass resolution quadrupole time-of-flight (Q-TOF) instrument (timsTOF flex MALDI2, Bruker Daltonics). Representative mass spectra for both polarities are shown in Figure S8. Abundant metabolites signal was detected in both positive- and negative-ion modes, including 196 and 431 assignments in positive- and negative-ion modes, respectively (Figure S6B, Tables S1 and S2). In the positive-ion mode, choline-containing phosphoglycerolipids, such as phosphatidylcholine and sphingomyelins (SMs), dominated the positive mode due to their naturally charged nature. Abundant metabolites were also detected in MALDI-MS in negative-ion mode. As expected, groups of metal chloride adduct were detected in the negative-ion mode, including sodium, calcium, potassium, magnesium, iron, and zinc as [MCln+1]^−^ and [m­(MCln) + Cl]^−^ and easily differentiate from other metabolites since its unique isotopic pattern^32^ (Figure S9 and Table S6). Using NEDC as a MALDI matrix not only achieves comprehensive coverage of small molecule metabolites and lipid species but also enables the detection of endogenous metal ion signals, which is challenging to attain with other matrices. In highly heterogeneous tissues, the presence of inorganic constituents cannot be ignored, as they typically play a significant role in metabolic homeostasis and physiological functions.^36,37^

The innovative workflow focuses on optimizing sample preparation for the MALDI MSI of rigid samples. Containment of the degrees of postsection cracks is achieved by altering the drying method and section thickness. A novel, contactless spin-flattening sample mounting technique was introduced to ensure sample surface flatness, which greatly improved the sample integrity and imaging quality. Finally, the imaging depth is improved in both metallomics and metabolomics dimensions by using the NEDC matrix. The current method demonstrates its superior performance in the MSI of fresh-frozen rigid tissue. To further explore its capability in imaging fresh-frozen heterogeneous tissue, we benchmarked it with the rodent cranium and knee joints.

### Spatial Mapping Molecular Heterogeneity of Mouse Skull

Cranium is a highly compartmentalized region in the body that contains tissue at different levels of moisture, lipids, and salts. A merged ion image of the mouse transverse section with detailed histological annotation is provided in Figure S10. This mouse cranium section contains the cerebellum, cranium, ear assembly, pharynx assembly, and a complex muscle system. Without proper embedding and the help of cryofilm, it is nearly impossible to section the film intactly. After the optical scan, the section was imaged with an ordinary mass spectrometry imaging workflow with a 30 μm raster width. Representative ion images of the mouse head section are shown in Figure 3. No obvious fissures and artifacts were observed in either ion images or optical scans, indicating good crack control (Figure 3). Accounting for improved sample smoothness, empty spots due to laser misalignment are also barely observed in the overlaid ion image (Figure 3I). Within the cerebrum, Sulfatide and PA (36:1) (Figure 3G,D) were found in the white matter of the cerebrum, while PS (40:6) and CAP (18:0) (Figure 3E,H) were found to be higher in intensity in the gray matter of the brain. Other ions such as calcium chloride ion and *N-*acetylgalactosamine sulfate (GalNAcS) were found in bone and cartilage, respectively (Figure 3B,C). The distribution of those metabolites is in line with the reported literature.^38,39^ A white dashed line enclosed in the inset in Figure 3I displays the zoom-in structure of cranium. A double-layer honeycomb-like bone structure was found with soft tissue in the vacated space. Right below the cranium, a thin layer of space between the cerebellum and cranium is annotated with dura and arachnoid compartment as a high level of Heme molecules was detected. Interestingly, four spherical areas with high intensity of ST (40:1) and PA (36:1) were found outside the brain (Figure 3D,G), and a magnified Figure 3I is shown in the orange dashed line enclosed insert. Based on the size of these structures, their chemical composition, and their relative anatomic position within the cranium, it can be inferred that they belong to the nervous system. Below the soft palate, a trachea-like structure is surrounded by plates with a high intensity of *N-*GalNAcS (Figure 3C,I). Based on the anatomic structure of the cranium, it should be the pharynx, which contains cartilage enriched with GalNAcS.^40^ It is worth noting that low levels of GalNAcS were also found in the white matter of the brain and intercranium space. GalNAcS was reported to involve protein glycosylation, synthesis of extracellular matrix molecules, and the substrate of GalNac-sulfotransferase for downstream metabolism and manufacturing.^41,42^ These anatomical structures resolved by mass spectrometry imaging demonstrate the high spatial resolution and high molecular coverage properties of the development method.

**Figure 3:**
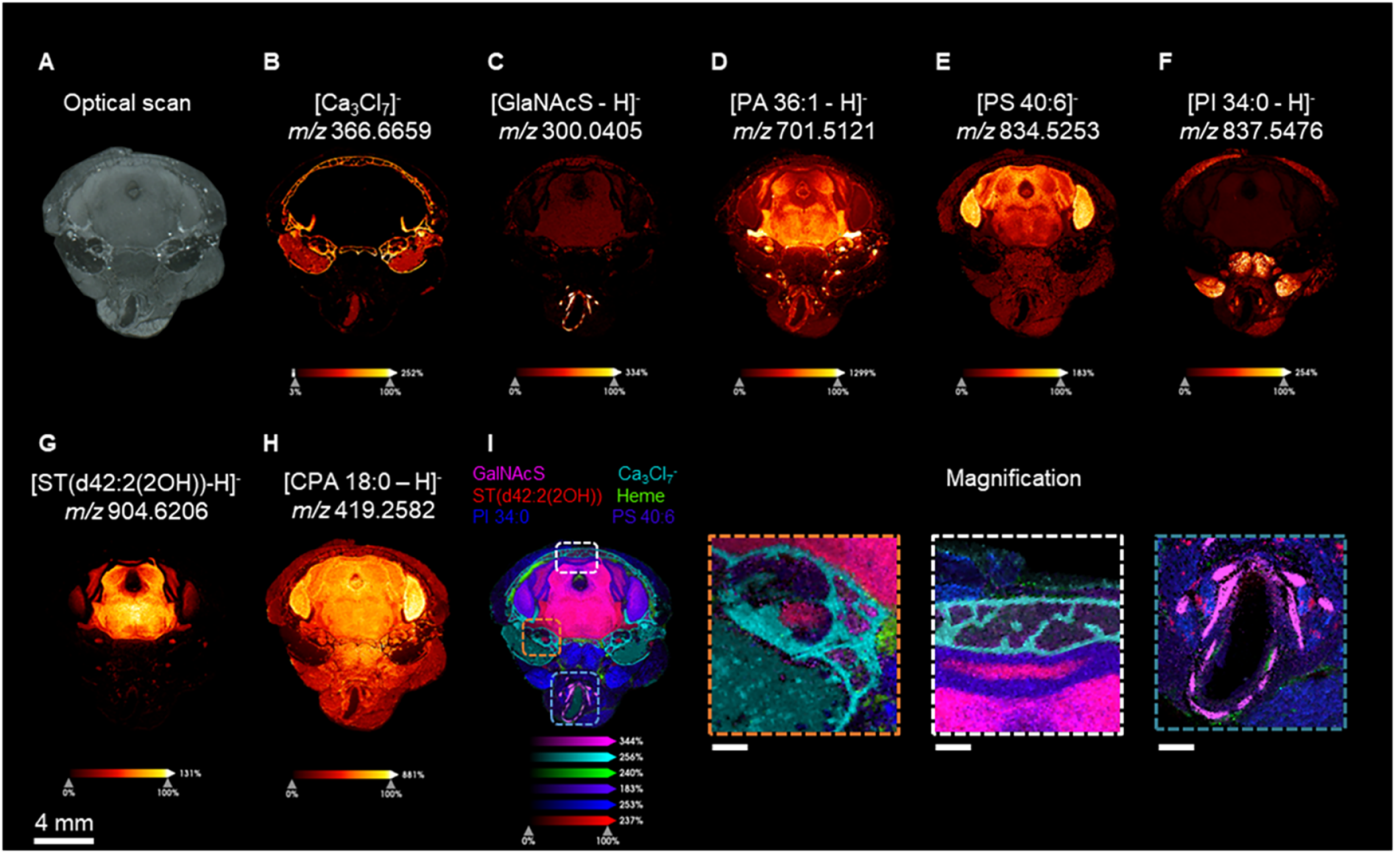
Representative ion images of the skull at 30 μm lateral resolution. (A) Optical scan of the imaged skull section. (B) Ion images of calcium cluster (Ca_3_Cl_7_^–^). (C) Distribution of *N*-acetylglucosamine sulfate in brain and cartilage. (D) Ion image of PA (36:1). (E) Ion image of PS (40:6). (F) Ion image of PI (34:0) enriched muscles. (G) Specific localization of ST­(d42:2­(2OH)). (H) Ion image of CAP (18:0). (I) Merge ion image of *N*-acetylglucosamine sulfate, Ca_3_Cl_7_^–^, ST­(d42:2­(2OH)), Heme, PI (34:0), and PS (40:6). Three insets display magnification of orange, white, and blue dashed lines enclose area of (I). A scale bar, representing 2 mm from (A–I), was displayed at the lower left corner of (I). For magnification inset, a scale bar displayed at the lower left corner represents 400, 400, and 700 μm for orange, white, and blue dashed lines enclosing ion image.

### Spatial Mapping of Metabolites and Inorganic Contents in Rat Joint

Through imaging metabolomics of the mouse cranium, the current workflow has demonstrated advantages in combating postsection cracks, improving molecular coverage, and enhancing imaging quality. We further applied this method to the knee joint of rats to explore previously overlooked functional molecules. Representative ion images generated are shown in Figures 4 and S11–S16. As expected, postsection cracks due to drying were not obvious in either ion images or the optical scan, further proving the effectiveness of our workflow in control artifacts (Figure 4A,B). PI (38:4) was the most abundant lipid found in all soft tissue regions except cartilage based on the averaged mass spectra shown in Figure S11A. The most intense signal in the cartilage region is colored orange in Figure S11A is PE (16:0/18:1), and it is highly expressed and specific to the cartilage region. As shown in Figure 4A, GalNAcS were also found to be highly expressed in the cartilage region, matching with what was observed in mouse cranium MSI. Interestingly, a panel of phosphoglycerolipids, regardless of the composition of head groups, with similar carbon acyl chain length and degree of saturation specifically expressed in cartilage (Figure S12B). Similarly, Lyso-phosphoglycerolipids LPE (18:1), which participated in the acryl chain remodeling process of phosphoglycerolipids, are also highly abundant in growth plate cartilage and absent in other regions (Figure S12B). This distinct molecular composition between cartilage and other regions suggests their different metabolic state. To further understand this interesting spatial preference of lipids and chain length, we performed tandem mass spectrometry for those highly spatially preferential metabolites to elucidate their acyl chain composition (Table S3). Most of those lipids contain palmitic acid or Oleic acid as their lipid building block. With one degree of unsaturation difference, PE (34:0) has no spatial preference in the cartilage area (Figure S11B). It is more intense in the femur and tibial bone marrow. Chondrocytes are the predominant cell type in the growth plate cartilage and regulate the growth of epiphyseal plates.^44^ Several reported studies indicate that saturated fatty acid plays an important role and is enriched in chondrocytes.^43–46^ It might support the proliferation of chondrocytes and provide mechanical features that absorb shock during motion. Another interesting finding is that a higher level of HSO_4_^–^ was found in the growth plate cartilage region (Figure S16). The high level of HSO_4_^–^ may be due to the presence of more sulfate salts in the region, or it could be the product of metastable ion containing sulfate (such as GalNAcS). Driven by its unique expression of saturated and monounsaturated lipids and high HSO_4_^–^ level, we have decided to further explore this area.

**Figure 4:**
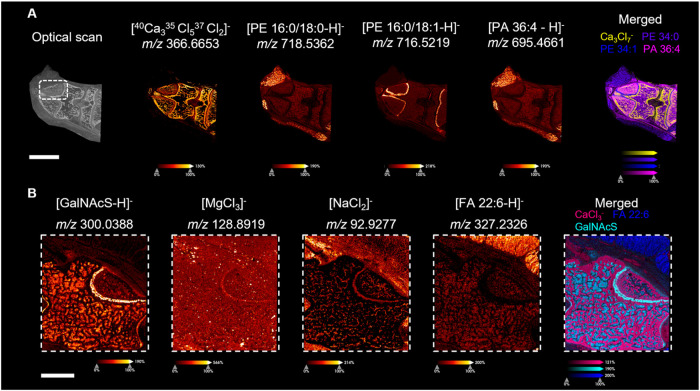
Representative ion images of small-molecular-weight metabolites and inorganic salt were obtained in negative-ion mode in the rat joint at 30 μm lateral resolution. All ion images except merged were pseudocolored with a fire color gradient. An individual color scale bar was displayed at the bottom of each ion image. The scale bar at the lower left corner of (A, B) represents 4 and 2 mm, respectively.

### Differential Expression of Metabolites in the Growth Plate in Youth and Adult Rat

To further investigate the molecular composition and structure of the growth plate cartilage, high spatial resolution MSI (10 μm) was performed at the epiphysis of the tibia bone (Figure 5). Compared with Figures 4A and 5E, the ion image of PE (34:1) acquired at higher resolution resolved the hollowed structure of the cartilage, which is not observed at lower resolution. Interestingly, several metabolites have very high spatial specificity, PE (34:1), N-GalNAcS, and six *N*-acetylated disaccharide sulfates (NADS) detected at the low *m*/*z* range, found primarily inside cartilage (Figures 5E and S18A). The structures of these NADS are very similar, except for changes in substituents and degree of unsaturation. Speculations on the structure of these NADS are shown in Figure S17A. Based on the assumption that they contain sulfate groups and have similar structures and comparable ionization efficiency. Thus, the relative spectral intensities of those NADSs could directly reflect their endogenous level. The unsaturated NADS2 is the most abundant NADS. NADS1 with an extra double bond has the least abundance in the proximal cartilage (Figure S17B). Although NADSs and PE (34:1) were almost exclusively detected in the growth plate cartilage, they occupied distinct regions. PE (34:1) is distributed at the distal side of the epiphysis, and NADSs are distributed at the proximal face of the epiphysis (Figures 5E and S18A). This distinct molecular localization might be associated with the different proliferative stages of chondrocytes.

**Figure 5:**
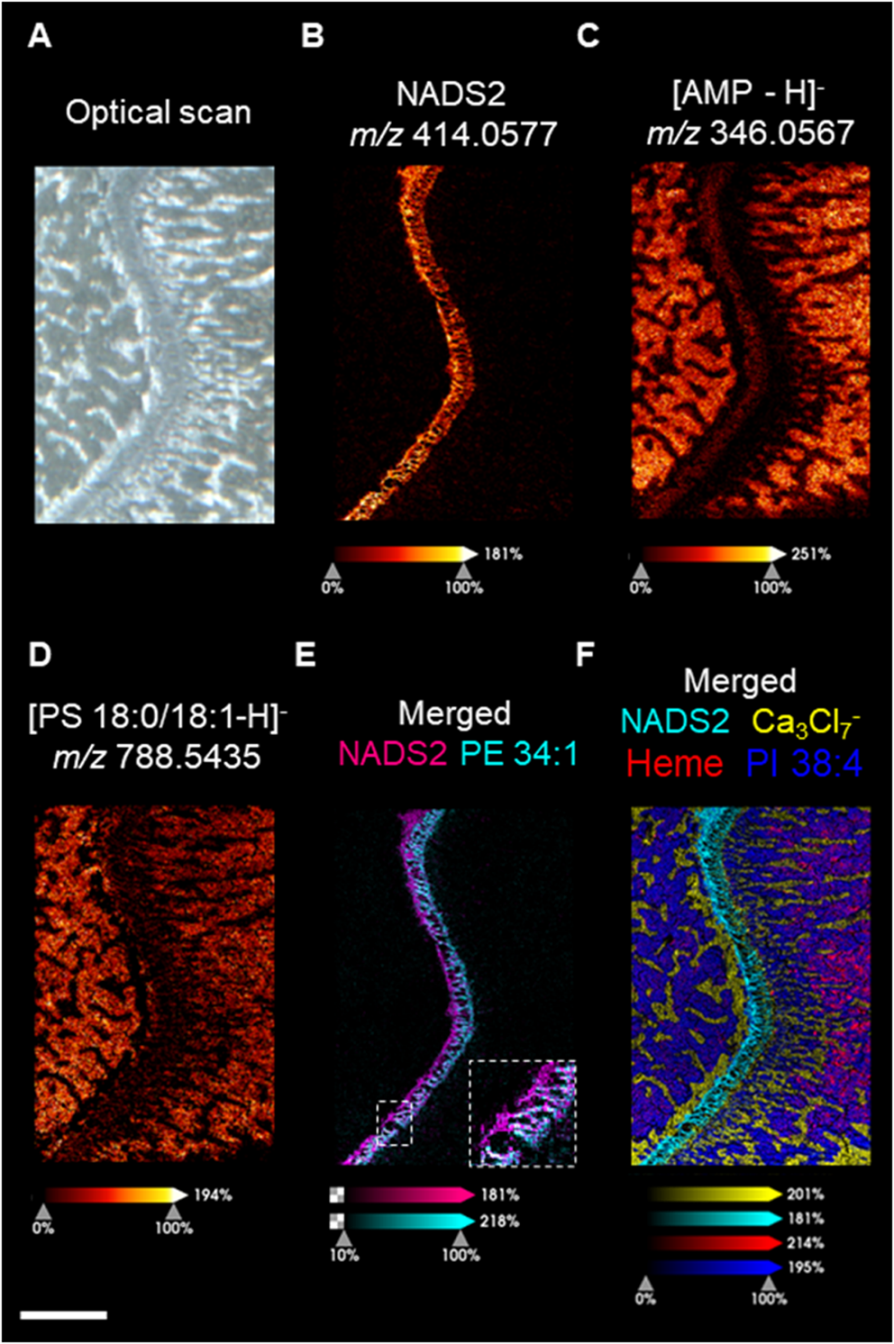
Representative ion images were obtained at a 10 μm lateral resolution. (A) Optical scan of growth plate cartilage. (B) Ion image of NADS2. (C) Ion image of AMP. (D) Ion image of PS (18:0/18:1). (E) Merged ion images of NADS2 and PE 34:1. (F) Merge ion image of NADS2, Calcium cluster, Heme, and PI 38:4. All ion images, except the merged one, were pseudocolored with a fire gradient scale indicating relative ion intensity. An individual color scale bar is displayed at the bottom of each ion image. A scale bar representing 1000 μm is displayed at the lower right of H.

To test this hypothesis, the epiphysis of rat tibia at different ages was imaged at 10 μm resolution (Figure 6). The RMS normalized intensity of most of the metabolites was not significantly altered in the tibial epiphysis of 4-week-old and 24-week-old rats. However, the normalized intensities of PE 36:1, PE 34:1, and six NADSs were found to be different at different ages (Figure S18B). The intensities of PE 36:1 and PE 34:1 were higher in the elder epiphysis, while NADSs were merely detected in the elder epiphysis. Based on the predicted structure of those NADSs and reported studies, those sulfated *N-*acetylglycosamine might be involved in extracellular matrix molecules, such as chondroitin, keratan, and Dermatan sulfate.^47,48^ Alteration of endogenous levels of sulfated *N-*acetylglycosamine might indicate a change in the extracellular matrix that provides a distinct microenvironment for cellular processes at different growth stages.

**Figure 6:**
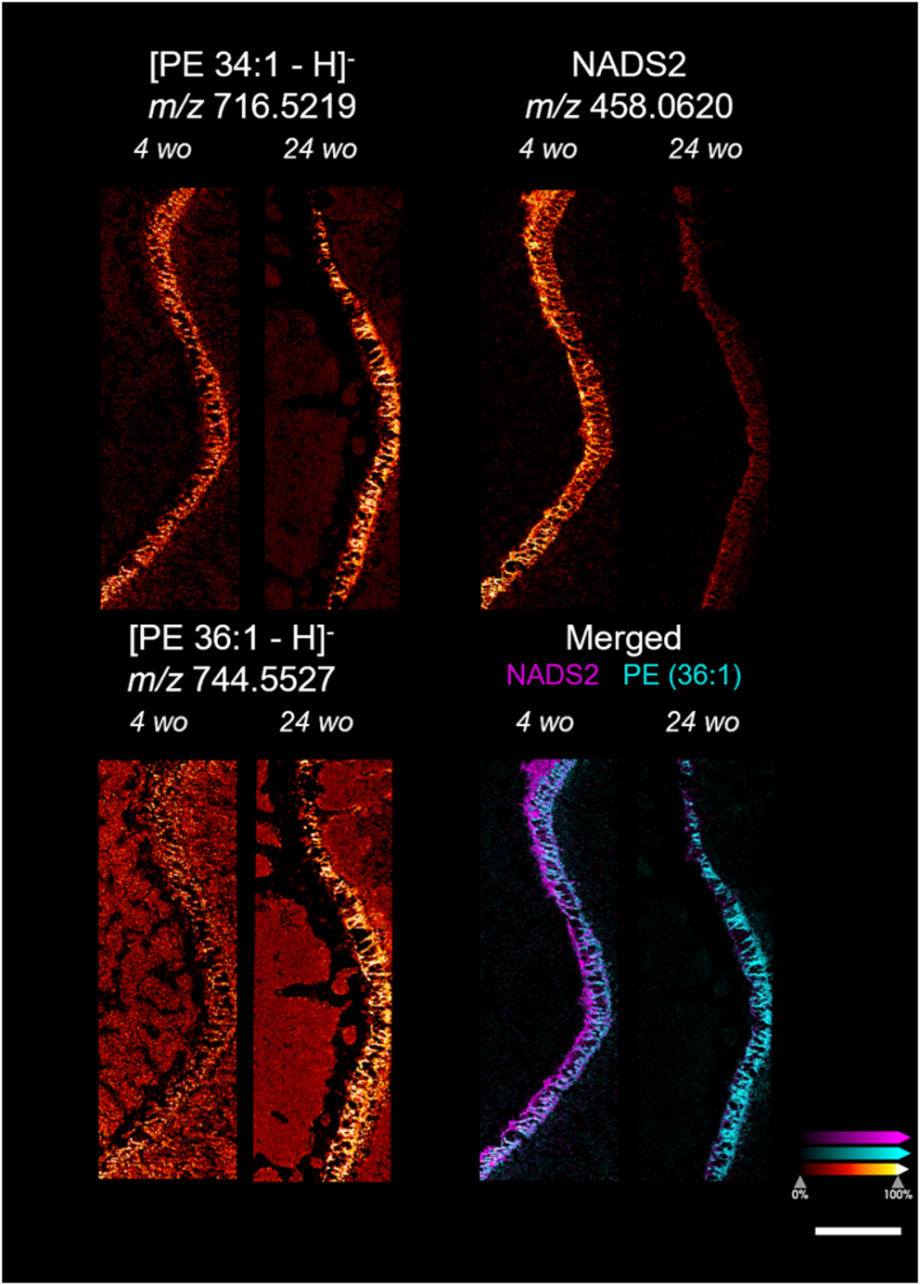
Representative images of the growth plate of 4-week-old (4 wo) and 24-week-old (24 wo) rats were obtained at 10 μm lateral resolution. PE (36:1), PE (34:1), NADS2, and merged ion images of NADS2 and PE (36:1) are shown. A scale bar is displayed in the lower left corner, representing 1000 μm. All ion images except the merged one were pseudocolored with a fire gradient scale indicating relative ion intensity at the lower left corner. An individual color scale bar is displayed for merged images at the lower left corner.

Collectively, based on our optimized method, its features contained postsection cracks and smooth surface topology that could support spatial resolution up to 10 μm. The high molecular feature of the current workflow allowed us to discover a range of metabolites, including saturated/unsaturated phosphatidylglycerols and sulfated *N*-acetylglucosamine, highly enriched in growth plate cartilage, possibly related to chondrocyte growth. Comparative imaging metabolomics of growth plate cartilage in different growth stages revealed a significant decrease in NADS in adult rats and upregulated PE 34:1, supporting our hypothesis that those molecules might involve and support the development of bone.

## Conclusions

In conclusion, our study presents an optimized workflow for imaging metabolomics of heterogeneous tissue that significantly reduces tissue cracking and artifacts in rigid samples by a combination of lyophilization and tailored section thickness. The implementation of lyophilization as a drying method has proven critical in preserving tissue integrity and enhancing the mass spectrometry imaging quality of fresh frozen rigid. Adopting the spin-flattening method, which uses centrifugation to remove excess mountant, ensures a contactless mounting and smooth surface. The use of the NEDC matrix has expanded molecular coverage, allowing for the detection of a broad spectrum of metabolites and lipid species as well as metal adducts, which is particularly beneficial for the analysis of mineralized rigid tissues. We apply our innovative workflow to comprehensively investigate metabolites and metal distribution in fresh frozen rodent skull and knee joints, assigning over 600 metabolites, including metal ions in negative-ion modes and ∼200 in positive-ion modes. Through our high coverage and resolution imaging workflow, the discovery of several bone-development-associated metabolites in growth plate cartilage further proves the robustness of our workflow. These methodological advancements collectively enhance MALDI MSI’s application in studying heterogeneous biological samples, offering improved resolution and reliability in molecular imaging. In the future, our workflow can potentially be applied to radiopharmaceutical research in bone metastasis due to its unique capabilities of imaging the metallome and metabolome. Simultaneous imaging of metabolome and metallome provides an opportunity to interrogate the interaction of mineralized bone, neoplastic tissue, and incorporated radiopharmaceuticals at the molecular level.

## Supplementary Material



## Notes

Additional experiment details, materials, methods, and figures; table of metabolites assigned in negative- and positive-ion mode; metabolites identified by tandem mass spectrometry; quantitative data on surface crack width and effect on bright-field scan and MALDI-MSI; quantitative data on surface flatness before and after spin-flattening; additional mass spectra of DHB, NEDC, and tissue; segmentation maps of knee joint and mouse skull; other ion images of metabolites in the knee joint; and ion images and mass spectra of NADS1-NADS6 in cartilage (PDF)
