# Supplementary material for: Comprehensive High-Spatial-Resolution Imaging Metabolomics Workflow for Heterogeneous Tissues
Source: Anal Chem. 2025 May 12;97(20):10561–9. doi: 10.1021/acs.analchem.4c05410 (PMC12120823; doi:10.1021/acs.analchem.4c05410)
Supplement: Supplementary file 1 [file ac4c05410_si_001.pdf]

# Comprehensive High Spatial Resolution Imaging Metabolomics Workflow for Heterogeneous Tissues

Xin Diao [1,2], Jianing Wang [1,3,*], Chengyi Xie [1,2], Leijian Chen [1,2], Thomas Ka-Yam LAM [1,2], Lin Zhu [1,2], Zongwei Cai [1,2,4,*]

[1] State Key Laboratory of Environmental and Biological Analysis, Hong Kong Baptist University, Hong Kong SAR 999077, China

[2] Department of Chemistry, Hong Kong Baptist University, Hong Kong SAR 999077, China

[3] School of Marine Science and Engineering, Hainan University, Haikou 570228, China

[4] Eastern Institute of Technology, Ningbo, Zhejiang 315200, China

[*] Corresponding Authors:

**Jianing Wang, Email: justin.wang.4in1@gmail.com**

**Zongwei Cai, Email: zwcai@hkbu.edu.hk**

**<u>Table of Content</u>**

Material and Methods: ....................................4

Table S1. Molecular assignments within five ppm relative error in negative ion mode. ..........6

Table S2. Molecular assignments within five ppm relative error in positive ion mode. .........22

Table S3. Molecular identification by tandem mass spectrometry ..........31

Table S4. Signal detected in DHB and NEDC in positive and negative ion mode, respectively. ....................................................................................................32

Table S5. The number of technical replicates performed to evaluate metabolome change at knee growth plate in different age. ....................................................................................34

Table S6. Relative mass error of all detected isotopic peaks, experimental isotopic distribution measured on tissue, metal chloride standards, and theoretical isotopic ratio. ......35

Figure S1. Detailed procedure of the proposed method. ....................................................39

Figure S2. Fresh frozen mouse skull sectioned at different thicknesses and dried by lyophilization. ..........................................................................................................40

Figure S3. The average intensity of fresh frozen mouse skull sectioned at different thicknesses and dried by lyophilization. ....................................................................41

Figure S4. Schematic diagram of centrifuge mounting. ......................................................42

Figure S5. The surface height of random points on tissue was determined by confocal microscopy with a 20X objective and 56 μm pinhole. ...................................................43

Figure S6. Representative averaged mass spectra in both positive and negative ion mode and merged ion image of the rat tibia epiphysis. ..................................................................44

Figure S7. Representative mass spectrum of 1,2 Dihydroxybenzoic acid (DHB), metabolome measured with DHB, (N-naphthyl) ethylenediamine hydrochloride (NEDC), and metabolome measured with NEDC. ..............................................................................................45

Figure S8. Representative mass spectrum was acquired using a high mass resolution Q-TOF instrument. ..........................................................................................................46

Figure S9. High-resolution mass spectra of seven metal chloride adducts were obtained in negative mode. .......................................................................................................47

Figure S10. Overlaid ion image of mouse cranium with anatomic annotation........................48

Figure S11. Averaged mass spectra and 30 μm lateral resolution ion images of cartilage, bone marrow, cortical bone, and muscle area.........................................................................................49

Figure S12. Representative 30 μm lateral resolution ion images of a rat joint generated by negative ion mode MALDI-MSI...............................................................................................50

Figure S13. Representative 30 μm lateral resolution ion images of rat joint generated by negative ion mode MALDI-MSI...............................................................................................52

Figure S14. Representative 30 μm lateral resolution ion images of polar metabolites (m/z < 500) in rat femur.......................................................................................................................54

Figure S15. Representative 30 μm lateral resolution ion images of polar metabolites (m/z < 500) in rat femur.......................................................................................................................56

Figure S16. Inorganic ions maps of rat femur measured at 30 μm lateral resolution. .............57

Figure S17. The proposed structures and mass spectra of NADS1 to NADS6. ......................58

Figure S18. Representative ion images and relative abundance of six NADSs are highly enriched in the growth plate cartilage region.........................................................................59

**Material and Methods:**

Methanol was purchased from VWR International (HPLC grade). Gelatin, 2,5- dihydroxy benzoic acid, sodium carboxymethyl cellulose, and *N*-(1-naphthyl) ethylenediamine dihydrochloride were purchased from Sigma Aldrich (St. Louis, USA). Cryofilm 3C 16UF was obtained from Section-Lab (Hiroshima, Japan). Indium tin oxide (ITO) coated glass slide was purchased from Delta Technologies (Loveland, USA). Adhesive for tissue-cyrofilm composite and ITO glass slide was brought from Kuretake (Nara, Japan, https://www.kuretakezig.us/product). Animals used in this work were purchased from the laboratory animal services center at the Chinese University of Hong Kong. All animal experiments were performed according to the guidelines for the use of experimental animals of HKBU and approved by HKBU Committee on the Use of Human & Animal Subjects in Teaching and Research.

Detailed Procedure:

As shown in the Figure S1, the method described in our work included five steps. A detailed workflow is summarized below, and the entire process before mass spectrometric measurement took around four hours. Before animal tissue collection, an embedding media solution of 10% gelatin (w/v) and 5% CMC (w/v) was prepared in Milli-Q water. Repeated heating (37 °C) and stirring were required to completely dissolve the gelatin and CMC. The solution was maintained at 37 °C until use; otherwise, it would solidify at room temperature. Once the tissue was ready, the tissue was placed in the mold in the preferred orientation, and the embedding media was poured into the mold. Then, the tissue was immediately snap-frozen in liquid nitrogen to prevent metabolite degradation and crystal formation. The Tissue block can be

stored at -80 °C until use. This work adopted Kawamoto's method, which uses cryofilm to support the sectioning. Briefly, the tissue block was trimmed until the expected cross-section was exposed. The adhesive side of the cryofilm was attached to the tissue block, and sectioning was performed at a constant speed. Then, the section was transferred into the lyophilizer (FreeZone, benchtop Freeze Dryer, 70020 Models) using a pre-chilled 50 mL conical tube and freeze-dried for at least two hours. After lyophilization, the embedding media surrounding the tissue should remain white. If it turned transparent, it indicated the section was thawed during the process. At this stage, the section could be stored in a vacuum-sealed plastic bag at -80 °C before the next step to prevent condensation and metabolite degradation. Before mounting, equilibrated the section to room temperature in the vacuum-sealed plastic bag. Excess ZIG 2-way glue was applied to the ITO glass to mount the section using the spin-flatten method. Then, with forceps, the section was transferred to the ITO, and one end of the section was attached with clean room tape to prevent the section from sliding off the ITO glass during spinning. Then, the ITO glass slide was transferred into a 50 mL conical tube in which the bottom was filled with foam and Kimwipes. The conical tube was centrifuged in a fixed angle rotor (Eppendorf 5910R with FA-6X50 rotor) at 7000 g for 10 minutes, and the slide was ready for matrix deposition. The matrix deposition and mass spectrometric measurement were described in the experimental section.

**Table S1. Molecular assignments within five ppm relative error in negative ion mode.**

| Assignment | Adduct form | Experimental *m/z* | Theoretical *m/z* | Relative error (ppm) |
|---|---|---|---|---|
| Gamma-Aminobutyric acid | $[M-H]^-$ | 102.0558 | 102.0561 | -2.9 |
| Taurine | $[M-H]^-$ | 124.0077 | 124.0074 | 2.4 |
| 2-Ketobutyric acid | $[M-H]^-$ | 101.0244 | 101.0244 | 0.0 |
| 3-Phosphoglyceric acid | $[M-H]^-$ | 184.9851 | 184.9857 | -3.2 |
| 3-Sulfinoalanine | $[M-H]^-$ | 152.0028 | 152.0023 | 3.3 |
| Acetylphosphate | $[M-H]^-$ | 138.9802 | 138.9802 | 0.0 |
| Adenosine monophosphate | $[M-H]^-$ | 346.0567 | 346.0558 | 2.6 |
| Alanine | $[M-H]^-$ | 88.0404 | 88.0404 | 0.0 |
| Citric acid | $[M-H]^-$ | 191.0204 | 191.0197 | 3.7 |
| Citric acid | $[M+Cl]^-$ | 226.9964 | 226.9964 | 0.0 |
| Creatine | $[M-H]^-$ | 130.0619 | 130.0622 | -2.3 |
| Decenedioic acid | $[M-H]^-$ | 199.0967 | 199.0976 | -4.5 |
| FA 16:0 | $[M-H]^-$ | 255.2335 | 255.2330 | 2.0 |
| FA 18:1 | $[M-H]^-$ | 281.2481 | 281.2486 | -1.8 |
| Dihydrothymine | $[M-H_2O-H]^-$ | 109.0402 | 109.0407 | -4.6 |
| Dimethyl 2-oxoglutarate | $[M-H]^-$ | 173.0448 | 173.0455 | -4.0 |
| Ethyl hydrogen sulfate | $[M-H_2O-H]^-$ | 106.9805 | 106.9808 | -2.8 |
| FA 18:0 | $[M-H]^-$ | 283.2635 | 283.2643 | -2.8 |
| FA 18:2;O | $[M-H]^-$ | 295.2289 | 295.2279 | 3.4 |
| FA 20:4 | $[M-H]^-$ | 303.2328 | 303.2330 | -0.7 |
| FA 20:2 | $[M-H]^-$ | 307.2634 | 307.2643 | -2.9 |
| FA 18:2 | $[M-H]^-$ | 279.2329 | 279.2330 | -0.4 |
| FA 20:1 | $[M-H]^-$ | 309.2791 | 309.2799 | -2.6 |
| FA 16:4;O2 | $[M+Cl]^-$ | 315.1367 | 315.1369 | -0.6 |
| FA 20:4;O | $[M-H]^-$ | 319.2273 | 319.2279 | -1.9 |

| | | | | |
|---|---|---|---|---|
| FA 22:7 | $[M-H]^-$ | 325.2173 | 325.2173 | 0.0 |
| FA 18:4;O | $[M+Cl]^-$ | 327.1722 | 327.1733 | -3.4 |
| FA 22:6 | $[M-H]^-$ | 327.2326 | 327.2330 | -1.2 |
| FA 20:3 | $[M-H]^-$ | 305.2499 | 305.2486 | 4.3 |
| FA 22:6;O | $[M-H]^-$ | 343.2263 | 343.2279 | -4.7 |
| FA 20:4 | $[M+Cl]^-$ | 339.2084 | 339.2096 | -3.5 |
| FA 22:5;O | $[M-H]^-$ | 345.2444 | 345.2435 | 2.6 |
| FA 20:6;O3 | $[M-H]^-$ | 347.1863 | 347.1864 | -0.3 |
| FA 20:5 | $[M-H]^-$ | 301.2169 | 301.2173 | -1.3 |
| FA 20:4;O | $[M+Cl]^-$ | 355.2037 | 355.2046 | -2.5 |
| FA 22:4 | $[M-H]^-$ | 331.2639 | 331.2643 | -1.2 |
| FA 20:1;O3 | $[M-H]^-$ | 357.2661 | 357.2647 | 3.9 |
| FA 22:5 | $[M-H]^-$ | 329.2485 | 329.2486 | -0.3 |
| FA 22:7 | $[M+Cl]^-$ | 361.1929 | 361.1940 | -3.0 |
| FA 22:6 | $[M+Cl]^-$ | 363.2089 | 363.2096 | -1.9 |
| FA 22:4 | $[M+Cl]^-$ | 367.2405 | 367.2409 | -1.1 |
| FA 20:2;O2 | $[M+Cl]^-$ | 375.2305 | 375.2308 | -0.8 |
| FA 22:5;O | $[M+Cl]^-$ | 381.2211 | 381.2202 | 2.4 |
| FA 28:3 | $[M-H]^-$ | 417.3741 | 417.3738 | 0.7 |
| FA 26:4 | $[M+Cl]^-$ | 423.3054 | 423.3035 | 4.5 |
| Fructose 1,6-bisphosphate | $[M-H]^-$ | 338.9885 | 338.9888 | -0.9 |
| Fructose-6-Phosphate | $[M-H]^-$ | 259.0224 | 259.0224 | 0.0 |
| Fructose-6-Phosphate | $[M-H_2O-H]^-$ | 241.0121 | 241.0119 | 0.8 |
| Glutamate | $[M-H]^-$ | 146.0453 | 146.0459 | -4.1 |
| Glutaric acid | $[M-H]^-$ | 131.0349 | 131.0350 | -0.8 |
| Glutathione | $[M-H]^-$ | 306.0765 | 306.0765 | 0.0 |
| Glyceraldehyde 3-phosphate | $[M-H]^-$ | 168.9899 | 168.9907 | -4.7 |
| Glycerol 3-phosphate | $[M-H_2O-H]^-$ | 152.9952 | 152.9958 | -3.9 |
| Glycerol 3-phosphate | $[M-H]^-$ | 171.0065 | 171.0064 | 0.6 |
| Glycerophosphoinositol | $[M-H_2O-H]^-$ | 315.0479 | 315.0487 | -2.5 |

| | | | | |
|---|---|---|---|---|
| Histamine | $[M-H]^-$ | 110.0722 | 110.0724 | -1.8 |
| Histidine | $[M-H]^-$ | 154.0625 | 154.0622 | 1.9 |
| Histidylalanine | $[M-H]^-$ | 225.0995 | 225.0993 | 0.9 |
| Inosinic acid | $[M-H]^-$ | 347.0395 | 347.0398 | -0.9 |
| inositol cyclic phosphate | $[M-H]^-$ | 241.0121 | 241.0119 | 0.8 |
| Methionyl-Glycine | $[M-H]^-$ | 205.0647 | 205.0652 | -2.4 |
| Methylisocitric acid | $[M-H]^-$ | 241.0121 | 241.0113 | 3.3 |
| myo-Inositol 1-phosphate/Fructose-6-Phosphate | $[M-H_2O-H]^-$ | 241.0121 | 241.0119 | 0.8 |
| Naphthalene-1,2-diol | $[M-H]^-$ | 159.0456 | 159.0452 | 2.5 |
| octanone | $[M+Cl]^-$ | 163.0894 | 163.0895 | -0.6 |
| O-Phosphoethanolamine | $[M-H]^-$ | 140.0116 | 140.0118 | -1.4 |
| Ornithine | $[M-H]^-$ | 131.0825 | 131.0826 | -0.8 |
| Oxo-tetradecanoic acid | $[M-H]^-$ | 241.1820 | 241.1809 | 4.6 |
| Phosphodimethylethanolamine | $[M-H]^-$ | 168.0432 | 168.0431 | 0.6 |
| Phosphonoacetate | $[M-H]^-$ | 138.9802 | 138.9802 | 0.0 |
| Pyrrole-2-carboxylic acid | $[M-H]^-$ | 110.0248 | 110.0248 | 0.0 |
| Pyruvic acid | $[M+Cl]^-$ | 122.9856 | 122.9854 | 1.6 |
| Ribose-5-phosphate | $[M-H_2O-H]^-$ | 211.0007 | 211.0013 | -2.8 |
| Succinimide | $[M-H]^-$ | 98.0244 | 98.0248 | -4.1 |
| Tetrahydropteridine | $[M+Cl]^-$ | 171.0446 | 171.0443 | 1.8 |
| Threonine | $[M-H_2O-H]^-$ | 100.0401 | 100.0404 | -3.0 |
| Tryptophan | $[M-H]^-$ | 203.0834 | 203.0826 | 3.9 |
| Tryptophan | $[M-H]^-$ | 203.0834 | 203.0826 | 3.9 |
| Uric acid | $[M-H]^-$ | 167.0219 | 167.0211 | 4.8 |
| Uridine 5'-monophosphate | $[M-H]^-$ | 323.0275 | 323.0286 | -3.4 |
| Cer 38:1;O | $[M+Cl]^-$ | 612.5471 | 612.5492 | -3.4 |
| Cer 40:0;O2 | $[M+Cl]^-$ | 658.5942 | 658.5911 | 4.7 |
| Cer 42:1;O | $[M+Cl]^-$ | 668.6145 | 668.6118 | 4.0 |

| | | | | |
|---|---|---|---|---|
| CerP 36:1;O2 | [M-H]$^-$ | 644.5014 | 644.5025 | -1.7 |
| CerP 42:1;O2 | [M-H]$^-$ | 728.5985 | 728.5964 | 2.9 |
| CerPE 36:0;O3 | [M+Cl]$^-$ | 741.5286 | 741.5319 | -4.5 |
| CerPE 36:1;O2 | [M-H]$^-$ | 687.5442 | 687.5447 | -0.7 |
| CerPE 36:1;O3 | [M-H]$^-$ | 703.5382 | 703.5396 | -2.0 |
| CerPE 36:3;O2 | [M-H]$^-$ | 683.5167 | 683.5134 | 4.8 |
| CerPE 38:1;O2 | [M-H]$^-$ | 715.5743 | 715.5760 | -2.4 |
| CerPE 38:2;O2 | [M-H]$^-$ | 713.5598 | 713.5603 | -0.7 |
| CerPE 38:3;O3 | [M-H]$^-$ | 727.5409 | 727.5396 | 1.8 |
| CerPE 40:1;O2 | [M-H]$^-$ | 743.6053 | 743.6073 | -2.7 |
| LPA 18:2 | [M-H]$^-$ | 433.2352 | 433.2361 | -2.1 |
| LPA 18:1 | [M-H]$^-$ | 435.2498 | 435.2517 | -4.4 |
| LPE O-16:1 | [M-H]$^-$ | 436.2817 | 436.2834 | -3.9 |
| LPA 18:0 | [M-H]$^-$ | 437.2660 | 437.2674 | -3.2 |
| FA 28:8 | [M+Cl]$^-$ | 443.2726 | 443.2722 | 0.9 |
| Hex2Cer 30:1;O2 | [M+Cl]$^-$ | 840.5260 | 840.5246 | 1.7 |
| FA 28:7 | [M+Cl]$^-$ | 445.2886 | 445.2879 | 1.6 |
| LPE 16:1 | [M-H]$^-$ | 450.2647 | 450.2626 | 4.7 |
| FA 28:4 | [M+Cl]$^-$ | 451.3327 | 451.3348 | -4.7 |
| LPA 16:0 | [M-H]$^-$ | 409.2362 | 409.2361 | 0.2 |
| LPE O-16:0;O | [M-H]$^-$ | 454.2923 | 454.2939 | -3.5 |
| LPG O-16:1 | [M-H]$^-$ | 467.2771 | 467.2779 | -1.7 |
| LPA 18:2 | [M+Cl]$^-$ | 469.2143 | 469.2128 | 3.2 |
| LPS O-16:1 | [M-H]$^-$ | 480.2727 | 480.2732 | -1.0 |
| LPE O-18:1;O | [M-H]$^-$ | 480.3074 | 480.3096 | -4.6 |
| LPA 20:3 | [M-H]$^-$ | 459.2506 | 459.2517 | -2.4 |
| LPA 20:4 | [M-H]$^-$ | 457.2353 | 457.2361 | -1.7 |
| LPA 22:6 | [M-H]$^-$ | 481.2351 | 481.2361 | -2.1 |
| LPG 16:1 | [M-H]$^-$ | 481.2578 | 481.2572 | 1.2 |
| LPG 16:0 | [M-H]$^-$ | 483.2736 | 483.2729 | 1.4 |

| | | | | |
|---|---|---|---|---|
| LPS 16:1 | $[M-H]^-$ | 494.2508 | 494.2525 | -3.4 |
| LPE 20:5 | $[M-H]^-$ | 498.2626 | 498.2626 | 0.0 |
| LPA 20:0 | $[M+Cl]^-$ | 501.2776 | 501.2754 | 4.4 |
| LPG O-16:1 | $[M+Cl]^-$ | 503.2569 | 503.2546 | 4.6 |
| LPE 18:4 | $[M+Cl]^-$ | 508.2253 | 508.2237 | 3.1 |
| LPS O-18:1 | $[M-H]^-$ | 508.3055 | 508.3045 | 2.0 |
| LPE 20:0 | $[M-H]^-$ | 508.3400 | 508.3409 | -1.8 |
| LPG 18:1 | $[M-H]^-$ | 509.2905 | 509.2885 | 3.9 |
| LPE 18:0 | $[M+Cl]^-$ | 516.2854 | 516.2863 | -1.7 |
| LPE 16:0 | $[M-H]^-$ | 452.2783 | 452.2783 | 0.0 |
| LPE O-18:1;O | $[M+Cl]^-$ | 516.2854 | 516.2863 | -1.7 |
| LPE 18:0 | $[M-H]^-$ | 480.3080 | 480.3096 | -3.3 |
| LPE 22:6 | $[M-H]^-$ | 524.2766 | 524.2783 | -3.2 |
| LPE 18:1 | $[M-H]^-$ | 478.2953 | 478.2939 | 2.9 |
| LPE 18:2 | $[M-H]^-$ | 476.2797 | 476.2783 | 2.9 |
| LPA 22:2 | $[M+Cl]^-$ | 525.2754 | 525.2754 | 0.0 |
| LPC 16:0 | $[M+Cl]^-$ | 530.3036 | 530.3019 | 3.2 |
| LPG O-18:1 | $[M+Cl]^-$ | 531.2856 | 531.2859 | -0.6 |
| LPE 20:1 | $[M-H]^-$ | 506.3239 | 506.3252 | -2.6 |
| LPE 20:5 | $[M+Cl]^-$ | 534.2417 | 534.2393 | 4.5 |
| LPG 20:2 | $[M-H]^-$ | 535.3045 | 535.3042 | 0.6 |
| LPG 18:3 | $[M+Cl]^-$ | 541.2364 | 541.2339 | 4.6 |
| LPE 22:4 | $[M-H]^-$ | 528.3084 | 528.3096 | -2.3 |
| LPE 20:0 | $[M+Cl]^-$ | 544.3190 | 544.3176 | 2.6 |
| LPS 20:3 | $[M-H]^-$ | 546.2822 | 546.2838 | -2.9 |
| LPS 20:1 | $[M-H]^-$ | 550.3138 | 550.3151 | -2.4 |
| LPS 18:4 | $[M+Cl]^-$ | 552.2149 | 552.2135 | 2.5 |
| LPS 18:1 | $[M+Cl]^-$ | 558.2619 | 558.2604 | 2.7 |
| LPG O-20:1 | $[M+Cl]^-$ | 559.3173 | 559.3172 | 0.2 |
| LPE O-18:0 | $[M-H]^-$ | 466.3292 | 466.3303 | -2.4 |

| | | | | |
|---|---|---|---|---|
| LPE O-18:1 | $[M-H]^-$ | 464.3149 | 464.3147 | 0.4 |
| LPE 24:1 | $[M-H]^-$ | 562.3889 | 562.3878 | 2.0 |
| LPG 22:2 | $[M-H]^-$ | 563.3355 | 563.3355 | 0.0 |
| LPE O-20:1 | $[M-H]^-$ | 492.3442 | 492.3460 | -3.7 |
| LPG 20:5 | $[M+Cl]^-$ | 565.2311 | 565.2339 | -5.0 |
| LPE 22:1 | $[M+Cl]^-$ | 570.3355 | 570.3332 | 4.0 |
| LPC O-20:1 | $[M+Cl]^-$ | 570.3688 | 570.3696 | -1.4 |
| LPS 22:4 | $[M-H]^-$ | 572.2973 | 572.2994 | -3.7 |
| LPS 22:2 | $[M-H]^-$ | 576.3300 | 576.3307 | -1.2 |
| LPC 20:4 | $[M+Cl]^-$ | 578.3003 | 578.3019 | -2.8 |
| LPG 22:0 | $[M-H]^-$ | 567.3641 | 567.3668 | -4.8 |
| LPC 20:3 | $[M+Cl]^-$ | 580.3197 | 580.3176 | 3.6 |
| LPS 22:0 | $[M-H]^-$ | 580.3616 | 580.3620 | -0.7 |
| LPE 24:6 | $[M+Cl]^-$ | 588.2857 | 588.2863 | -1.0 |
| LPI O-16:1 | $[M+Cl]^-$ | 591.2708 | 591.2707 | 0.2 |
| LPI O-16:0 | $[M+Cl]^-$ | 593.2853 | 593.2863 | -1.7 |
| LPI 16:0 | $[M-H]^-$ | 571.2897 | 571.2889 | 1.4 |
| LPI 18:0 | $[M-H]^-$ | 599.3186 | 599.3202 | -2.7 |
| LPI 18:0 | $[M-H_2O-H]^-$ | 581.3069 | 581.3091 | -3.8 |
| LPE 24:1 | $[M+Cl]^-$ | 598.3643 | 598.3645 | -0.3 |
| LPI 18:0 | $[M-H]^-$ | 599.3193 | 599.3202 | -1.5 |
| LPC 22:6 | $[M+Cl]^-$ | 602.3035 | 602.3019 | 2.7 |
| LPI 20:4 | $[M-H]^-$ | 619.2884 | 619.2889 | -0.8 |
| LPC 22:5 | $[M+Cl]^-$ | 604.3158 | 604.3176 | -3.0 |
| LPC 22:4 | $[M+Cl]^-$ | 606.3329 | 606.3332 | -0.5 |
| LPC 22:2 | $[M+Cl]^-$ | 610.3656 | 610.3645 | 1.8 |
| LPC 22:1 | $[M+Cl]^-$ | 612.3773 | 612.3802 | -4.7 |
| LPI O-16:0 | $[M-H]^-$ | 557.3081 | 557.3096 | -2.7 |
| LPC 22:0 | $[M+Cl]^-$ | 614.3981 | 614.3958 | 3.7 |
| LPS 22:0 | $[M+Cl]^-$ | 616.3390 | 616.3387 | 0.5 |

| | | | | |
|---|---|---|---|---|
| LPI 20:5 | $[M-H]^-$ | 617.2705 | 617.2732 | -4.4 |
| LPS O-22:0;O | $[M+Cl]^-$ | 618.3531 | 618.3543 | -1.9 |
| LPI O-18:1 | $[M+Cl]^-$ | 619.3024 | 619.3020 | 0.6 |
| LPS 18:3 | $[M+Cl]^-$ | 554.2313 | 554.2291 | 4.0 |
| LPI 20:1 | $[M-H]^-$ | 625.3349 | 625.3358 | -1.4 |
| LPI 22:6 | $[M-H]^-$ | 643.2915 | 643.2889 | 4.0 |
| LPI 22:4 | $[M-H]^-$ | 647.3221 | 647.3202 | 2.9 |
| LPI 20:1 | $[M+Cl]^-$ | 661.3124 | 661.3125 | -0.2 |
| PA 34:6 | $[M-H]^-$ | 663.4012 | 663.4031 | -2.9 |
| LPS O-28:1;O | $[M-H]^-$ | 664.4542 | 664.4559 | -2.6 |
| PA 34:2 | $[M-H]^-$ | 671.4642 | 671.4657 | -2.2 |
| PA 34:1 | $[M-H]^-$ | 673.4795 | 673.4814 | -2.8 |
| PA 34:0 | $[M-H]^-$ | 675.4947 | 675.4970 | -3.4 |
| DG 38:5 | $[M+Cl]^-$ | 677.4941 | 677.4917 | 3.5 |
| PE 32:5 | $[M-H]^-$ | 680.4266 | 680.4297 | -4.6 |
| PA 32:0 | $[M-H]^-$ | 647.4645 | 647.4657 | -1.9 |
| PA 32:1 | $[M+Cl]^-$ | 681.4253 | 681.4268 | -2.2 |
| PA 32:2 | $[M-H]^-$ | 643.4320 | 643.4344 | -3.7 |
| SM 34:1;O2 | $[M-CH3]^-$ | 687.5442 | 687.5447 | -0.7 |
| LPI 22:1 | $[M+Cl]^-$ | 689.3419 | 689.3438 | -2.8 |
| PA O-34:4 | $[M+Cl]^-$ | 689.4347 | 689.4319 | 4.1 |
| PA 34:3 | $[M-H]^-$ | 669.4474 | 669.4501 | -4.0 |
| PA 34:5 | $[M+Cl]^-$ | 701.3953 | 701.3955 | -0.3 |
| PA 36:6 | $[M-H]^-$ | 691.4359 | 691.4344 | 2.2 |
| PA 34:6 | $[M+Cl]^-$ | 699.3823 | 699.3798 | 3.6 |
| PA 36:0 | $[M+Cl]^-$ | 739.5051 | 739.5050 | 0.1 |
| PA 36:1 | $[M-H]^-$ | 701.5121 | 701.5127 | -0.9 |
| PA O-34:3 | $[M+Cl]^-$ | 691.4508 | 691.4475 | 4.8 |
| PA 36:3 | $[M-H]^-$ | 697.4782 | 697.4814 | -4.6 |
| PA 36:4 | $[M-H]^-$ | 695.4661 | 695.4657 | 0.6 |

| | | | | |
|---|---|---|---|---|
| PE O-34:5 | $[M-H]^-$ | 694.4838 | 694.4817 | 3.0 |
| PA 36:5 | $[M-H]^-$ | 693.4487 | 693.4501 | -2.0 |
| PA 36:2 | $[M-H]^-$ | 699.4946 | 699.4970 | -3.4 |
| PA O-38:7 | $[M-H]^-$ | 703.4730 | 703.4708 | 3.1 |
| PA O-38:6 | $[M-H]^-$ | 705.4842 | 705.4865 | -3.3 |
| PG 32:5 | $[M-H]^-$ | 711.4222 | 711.4243 | -3.0 |
| PA O-36:6 | $[M+Cl]^-$ | 713.4296 | 713.4319 | -3.2 |
| SM 36:2;O2 | $[M-CH3]^-$ | 713.5598 | 713.5603 | -0.7 |
| PA 40:0 | $[M+Cl]^-$ | 795.5714 | 795.5676 | 4.8 |
| PC 32:2 | $[M-CH3]^-$ | 714.5052 | 714.5079 | -3.8 |
| PE 34:2 | $[M-H]^-$ | 714.5052 | 714.5079 | -3.8 |
| PA 40:4 | $[M-H]^-$ | 751.5251 | 751.5283 | -4.3 |
| SM 36:1;O2 | $[M-CH3]^-$ | 715.5743 | 715.5760 | -2.4 |
| PC 32:1 | $[M-CH3]^-$ | 716.5219 | 716.5236 | -2.4 |
| PA 40:6 | $[M-H]^-$ | 747.4949 | 747.4970 | -2.8 |
| PA 40:7 | $[M-H]^-$ | 745.4804 | 745.4814 | -1.3 |
| PE 34:1 | $[M-H]^-$ | 716.5219 | 716.5236 | -2.4 |
| PA 38:7 | $[M-H]^-$ | 717.4489 | 717.4501 | -1.7 |
| PA 42:0 | $[M+Cl]^-$ | 823.6007 | 823.5989 | 2.2 |
| PA 42:2 | $[M+Cl]^-$ | 819.5703 | 819.5676 | 3.3 |
| PA O-38:0 | $[M-H]^-$ | 717.5789 | 717.5804 | -2.1 |
| PC 32:0 | $[M-CH3]^-$ | 718.5362 | 718.5392 | -4.2 |
| PA O-34:1 | $[M-H]^-$ | 659.4996 | 659.5021 | -3.8 |
| PE 34:0 | $[M-H]^-$ | 718.5362 | 718.5392 | -4.2 |
| PA 38:6 | $[M-H]^-$ | 719.4642 | 719.4657 | -2.1 |
| PA O-36:0 | $[M-H]^-$ | 689.5464 | 689.5491 | -3.9 |
| PA O-36:2 | $[M-H]^-$ | 685.5194 | 685.5178 | 2.3 |
| PA 38:5 | $[M-H]^-$ | 721.4779 | 721.4814 | -4.9 |
| PC O-34:5 | $[M-CH3]^-$ | 722.5107 | 722.5130 | -3.2 |
| PA O-38:1 | $[M+Cl]^-$ | 751.5423 | 751.5414 | 1.2 |

| | | | | |
|---|---|---|---|---|
| PA O-38:3 | $[M+Cl]^-$ | 747.5133 | 747.5101 | 4.3 |
| PA O-38:5 | $[M-H]^-$ | 707.5002 | 707.5021 | -2.7 |
| PE O-36:5 | $[M-H]^-$ | 722.5107 | 722.5130 | -3.2 |
| PA 38:4 | $[M-H]^-$ | 723.4956 | 723.4970 | -1.9 |
| PC O-34:3 | $[M-CH3]^-$ | 726.5436 | 726.5443 | -1.0 |
| PA O-40:0 | $[M+Cl]^-$ | 781.5906 | 781.5884 | 2.8 |
| PA O-40:1 | $[M+Cl]^-$ | 779.5764 | 779.5727 | 4.7 |
| PE O-36:3 | $[M-H]^-$ | 726.5436 | 726.5443 | -1.0 |
| PA O-40:5 | $[M+Cl]^-$ | 771.5132 | 771.5101 | 4.0 |
| PC O-34:1 | $[M-CH3]^-$ | 730.5729 | 730.5756 | -3.7 |
| PE O-36:1 | $[M-H]^-$ | 730.5729 | 730.5756 | -3.7 |
| PA 36:4 | $[M+Cl]^-$ | 731.4392 | 731.4424 | -4.4 |
| SM 34:1;O2 | $[M+Cl]^-$ | 737.5337 | 737.5370 | -4.5 |
| PS O-34:5 | $[M-H]^-$ | 738.4706 | 738.4716 | -1.4 |
| PC 34:3 | $[M-CH3]^-$ | 740.5211 | 740.5236 | -3.4 |
| PE 36:3 | $[M-H]^-$ | 740.5211 | 740.5236 | -3.4 |
| PA O-38:6 | $[M+Cl]^-$ | 741.4606 | 741.4632 | -3.5 |
| PC 34:2 | $[M-CH3]^-$ | 742.5373 | 742.5392 | -2.6 |
| PE 36:2 | $[M-H]^-$ | 742.5373 | 742.5392 | -2.6 |
| PA 40:8 | $[M-H]^-$ | 743.4655 | 743.4657 | -0.3 |
| SM 38:1;O2 | $[M-CH3]^-$ | 743.6053 | 743.6073 | -2.7 |
| PE 34:5 | $[M+Cl]^-$ | 744.4380 | 744.4377 | 0.4 |
| PE 34:4 | $[M+Cl]^-$ | 746.4508 | 746.4533 | -3.3 |
| PG 32:5 | $[M+Cl]^-$ | 747.4000 | 747.4009 | -1.2 |
| PA 40:5 | $[M-H]^-$ | 749.5104 | 749.5127 | -3.1 |
| PS 34:6 | $[M-H]^-$ | 750.4338 | 750.4352 | -1.9 |
| PC O-36:5 | $[M-CH3]^-$ | 750.5412 | 750.5443 | -4.1 |
| PE O-38:5 | $[M-H]^-$ | 750.5412 | 750.5443 | -4.1 |
| PG O-36:6 | $[M-H]^-$ | 751.4916 | 751.4920 | -0.5 |
| CerPE 37:1;O3 | $[M+Cl]^-$ | 753.5334 | 753.5319 | 2.0 |

| | | | | |
|---|---|---|---|---|
| DG 44:8 | $[M+Cl]^-$ | 755.5383 | 755.5387 | -0.5 |
| PE O-36:6 | $[M+Cl]^-$ | 756.4775 | 756.4741 | 4.5 |
| PS O-32:0 | $[M+Cl]^-$ | 756.4947 | 756.4952 | -0.7 |
| PC 32:5 | $[M+Cl]^-$ | 758.4552 | 758.4533 | 2.5 |
| PC 32:4 | $[M+Cl]^-$ | 760.4659 | 760.4690 | -4.1 |
| PS 32:4 | $[M+Cl]^-$ | 762.4095 | 762.4118 | -3.0 |
| PC 36:6 | $[M-CH3]^-$ | 762.5062 | 762.5079 | -2.2 |
| PE 38:6 | $[M-H]^-$ | 762.5062 | 762.5079 | -2.2 |
| PE O-38:7;O | $[M-H]^-$ | 762.5062 | 762.5079 | -2.2 |
| PG 36:7 | $[M-H]^-$ | 763.4552 | 763.4556 | -0.5 |
| PA 38:2 | $[M+Cl]^-$ | 763.5084 | 763.5050 | 4.5 |
| PC 36:5 | $[M-CH3]^-$ | 764.5206 | 764.5236 | -3.9 |
| PE O-38:6;O | $[M-H]^-$ | 764.5206 | 764.5236 | -3.9 |
| SM 36:1;O2 | $[M+Cl]^-$ | 765.5683 | 765.5683 | 0.0 |
| PE 32:0 | $[M-H]^-$ | 690.5049 | 690.5079 | -4.3 |
| PC 36:4 | $[M-CH3]^-$ | 766.5370 | 766.5392 | -2.9 |
| PE 32:5 | $[M+Cl]^-$ | 716.4077 | 716.4064 | 1.8 |
| PE O-38:5;O | $[M-H]^-$ | 766.5370 | 766.5392 | -2.9 |
| PG 36:5 | $[M-H]^-$ | 767.4878 | 767.4869 | 1.2 |
| PA O-40:6 | $[M+Cl]^-$ | 769.4972 | 769.4945 | 3.5 |
| PC 36:2 | $[M-CH3]^-$ | 770.5691 | 770.5705 | -1.8 |
| PE 38:2 | $[M-H]^-$ | 770.5691 | 770.5705 | -1.8 |
| PE 36:0 | $[M-H]^-$ | 746.5680 | 746.5705 | -3.3 |
| PE 36:1 | $[M-H]^-$ | 744.5527 | 744.5549 | -3.0 |
| PE 36:5 | $[M+Cl]^-$ | 772.4701 | 772.4690 | 1.4 |
| PG 34:6 | $[M+Cl]^-$ | 773.4137 | 773.4166 | -3.7 |
| PE 36:4 | $[M-H]^-$ | 738.5063 | 738.5079 | -2.2 |
| PE 36:4 | $[M+Cl]^-$ | 774.4825 | 774.4846 | -2.7 |
| PG 36:2 | $[M-H]^-$ | 773.5304 | 773.5338 | -4.4 |
| PE 38:1 | $[M-H]^-$ | 772.5863 | 772.5862 | 0.1 |

| | | | | |
|---|---|---|---|---|
| PE 38:2 | [M-H]- | 770.5691 | 770.5705 | -1.8 |
| PS O-34:5 | [M+Cl]- | 774.4502 | 774.4482 | 2.6 |
| PE 38:3 | [M-H]- | 768.5545 | 768.5549 | -0.5 |
| PE 38:4 | [M-H]- | 766.5390 | 766.5392 | -0.3 |
| PC O-38:7 | [M-CH3]- | 774.5414 | 774.5443 | -3.7 |
| PE 38:5 | [M-H]- | 764.5232 | 764.5236 | -0.5 |
| PE 38:5 | [M+Cl]- | 800.4964 | 800.5003 | -4.9 |
| PG 36:1 | [M-H]- | 775.5463 | 775.5495 | -4.1 |
| PA 40:9 | [M+Cl]- | 777.4247 | 777.4268 | -2.7 |
| PG 34:4 | [M+Cl]- | 777.4494 | 777.4479 | 1.9 |
| PA O-40:2 | [M+Cl]- | 777.5601 | 777.5571 | 3.9 |
| PE 38:6;O | [M-H]- | 778.5024 | 778.5029 | -0.6 |
| PE 40:4 | [M-H]- | 794.5668 | 794.5705 | -4.7 |
| PC O-38:5 | [M-CH3]- | 778.5739 | 778.5756 | -2.2 |
| PE 38:5;O | [M-H]- | 780.5202 | 780.5185 | 2.2 |
| PE 40:7 | [M-H]- | 788.5237 | 788.5236 | 0.1 |
| PE 40:7 | [M+Cl]- | 824.4976 | 824.5003 | -3.3 |
| PE 40:8 | [M-H]- | 786.5072 | 786.5079 | -0.9 |
| PE O-38:7 | [M+Cl]- | 782.4916 | 782.4897 | 2.4 |
| PE 42:10 | [M+Cl]- | 846.4888 | 846.4846 | 5.0 |
| PC 36:4;O | [M-CH3]- | 782.5303 | 782.5342 | -5.0 |
| PE 38:4;O | [M-H]- | 782.5303 | 782.5342 | -5.0 |
| PG 34:1 | [M+Cl]- | 783.4959 | 783.4948 | 1.4 |
| PE O-34:0 | [M-H]- | 704.5590 | 704.5600 | -1.4 |
| PE O-34:1 | [M-H]- | 702.5438 | 702.5443 | -0.7 |
| PE O-34:3 | [M-H]- | 698.5112 | 698.5130 | -2.6 |
| PC 34:6 | [M+Cl]- | 784.4678 | 784.4690 | -1.5 |
| PC 38:8 | [M-CH3]- | 786.5070 | 786.5079 | -1.1 |
| PE O-36:2 | [M-H]- | 728.5575 | 728.5600 | -3.4 |
| PA 40:4 | [M+Cl]- | 787.5068 | 787.5050 | 2.3 |

| | | | | |
|---|---|---|---|---|
| PE O-36:4 | $[M-H]^-$ | 724.5270 | 724.5287 | -2.3 |
| PC 38:7 | $[M-CH3]^-$ | 788.5229 | 788.5236 | -0.9 |
| PE 40:7 | $[M-H]^-$ | 788.5229 | 788.5236 | -0.9 |
| PE O-38:4 | $[M-H]^-$ | 752.5564 | 752.5600 | -4.8 |
| PC 38:6 | $[M-CH3]^-$ | 790.5372 | 790.5392 | -2.5 |
| PE 40:6 | $[M-H]^-$ | 790.5372 | 790.5392 | -2.5 |
| PE O-38:6 | $[M-H]^-$ | 748.5275 | 748.5287 | -1.6 |
| PE O-40:7;O | $[M-H]^-$ | 790.5372 | 790.5392 | -2.5 |
| PA 40:1 | $[M+Cl]^-$ | 793.5528 | 793.5520 | 1.0 |
| PE O-38:7 | $[M-H]^-$ | 746.5109 | 746.5130 | -2.8 |
| PC 38:4 | $[M-CH3]^-$ | 794.5667 | 794.5705 | -4.8 |
| PG O-36:2 | $[M+Cl]^-$ | 795.5304 | 795.5312 | -1.0 |
| PE O-40:5 | $[M-H]^-$ | 778.5739 | 778.5756 | -2.2 |
| PE 38:7 | $[M+Cl]^-$ | 796.4675 | 796.4690 | -1.9 |
| PE O-40:7 | $[M-H]^-$ | 774.5414 | 774.5443 | -3.7 |
| PC 36:5;O2 | $[M-CH3]^-$ | 796.5162 | 796.5134 | 3.5 |
| PE O-40:8 | $[M-H]^-$ | 772.5257 | 772.5287 | -3.9 |
| PE O-40:8 | $[M+Cl]^-$ | 808.5082 | 808.5054 | 3.5 |
| PG O-36:1 | $[M+Cl]^-$ | 797.5471 | 797.5469 | 0.3 |
| SM 42:2;O2 | $[M-CH3]^-$ | 797.6504 | 797.6542 | -4.8 |
| PG 36:7 | $[M+Cl]^-$ | 799.4286 | 799.4322 | -4.5 |
| PA 44:8 | $[M-H]^-$ | 799.5286 | 799.5283 | 0.4 |
| PE O-38:6;O | $[M+Cl]^-$ | 800.4971 | 800.5003 | -4.0 |
| PS 38:8 | $[M-H]^-$ | 802.4640 | 802.4665 | -3.1 |
| PS O-36:4 | $[M+Cl]^-$ | 804.4925 | 804.4952 | -3.4 |
| PG 36:0 | $[M+Cl]^-$ | 813.5437 | 813.5418 | 2.3 |
| PS 38:6 | $[M-H]^-$ | 806.4941 | 806.4978 | -4.6 |
| PG 36:1 | $[M-H]^-$ | 775.5477 | 775.5495 | -2.3 |
| PG O-40:5 | $[M-H]^-$ | 809.5705 | 809.5702 | 0.4 |
| PS 36:8 | $[M+Cl]^-$ | 810.4101 | 810.4118 | -2.1 |

| | | | | |
|---|---|---|---|---|
| PG 36:2 | $[M+Cl]^-$ | 809.5136 | 809.5105 | 3.8 |
| PG 36:3 | $[M-H_2O-H]^-$ | 753.5039 | 753.5076 | -4.9 |
| PG 36:4 | $[M-H]^-$ | 769.4995 | 769.5025 | -3.9 |
| PS 38:4 | $[M-H]^-$ | 810.5256 | 810.5291 | -4.3 |
| PG 36:1 | $[M+Cl]^-$ | 811.5243 | 811.5261 | -2.2 |
| PE O-40:6 | $[M+Cl]^-$ | 812.5381 | 812.5367 | 1.7 |
| PA 42:4 | $[M+Cl]^-$ | 815.5386 | 815.5363 | 2.8 |
| PG 38:3 | $[M+Cl]^-$ | 835.5294 | 835.5261 | 3.9 |
| PG 38:5 | $[M+Cl]^-$ | 831.4948 | 831.4948 | 0.0 |
| PS 38:1 | $[M-H]^-$ | 816.5741 | 816.5760 | -2.3 |
| PC 36:2 | $[M+Cl]^-$ | 820.5595 | 820.5629 | -4.1 |
| PG 40:3 | $[M+Cl]^-$ | 863.5563 | 863.5574 | -1.3 |
| PI O-34:1 | $[M-H]^-$ | 821.5566 | 821.5549 | 2.1 |
| PE 40:8 | $[M+Cl]^-$ | 822.4861 | 822.4846 | 1.8 |
| PG 38:9 | $[M+Cl]^-$ | 823.4354 | 823.4322 | 3.9 |
| PG 42:4 | $[M+Cl]^-$ | 889.5712 | 889.5731 | -2.1 |
| PC O-38:7 | $[M+Cl]^-$ | 824.5401 | 824.5367 | 4.1 |
| PS O-38:5 | $[M+Cl]^-$ | 830.5142 | 830.5108 | 4.1 |
| PE 40:3 | $[M+Cl]^-$ | 832.5588 | 832.5629 | -4.9 |
| PS 40:6 | $[M-H]^-$ | 834.5253 | 834.5291 | -4.6 |
| PI 34:1 | $[M-H]^-$ | 835.5309 | 835.5342 | -3.9 |
| PG 38:2 | $[M+Cl]^-$ | 837.5432 | 837.5418 | 1.7 |
| PG O-42:5 | $[M-H]^-$ | 837.6037 | 837.6015 | 2.6 |
| PG O-36:5 | $[M-H]^-$ | 753.5041 | 753.5076 | -4.6 |
| PE O-42:7 | $[M+Cl]^-$ | 838.5535 | 838.5523 | 1.4 |
| PC 38:6 | $[M+Cl]^-$ | 840.5303 | 840.5316 | -1.5 |
| PE O-42:6 | $[M+Cl]^-$ | 840.5662 | 840.5680 | -2.1 |
| PI O-36:4 | $[M-H]^-$ | 843.5422 | 843.5393 | 3.4 |
| PI 30:3 | $[M-H]^-$ | 775.4423 | 775.4403 | 2.6 |
| PI 32:0 | $[M-H]^-$ | 809.5197 | 809.5186 | 1.4 |

| | | | | |
|---|---|---|---|---|
| PI 32:1 | [M-H]- | 807.5017 | 807.5029 | -1.5 |
| PC 38:4 | [M+Cl]- | 844.5592 | 844.5629 | -4.4 |
| PG O-40:4 | [M+Cl]- | 847.5589 | 847.5625 | -4.2 |
| PI 34:2 | [M-H]- | 833.5179 | 833.5186 | -0.8 |
| PI 34:3 | [M-H]- | 831.4999 | 831.5029 | -3.6 |
| PE 42:9 | [M+Cl]- | 848.4978 | 848.5003 | -2.9 |
| PI O-34:5 | [M+Cl]- | 849.4651 | 849.4690 | -4.6 |
| PI 34:5 | [M-H]- | 827.4720 | 827.4716 | 0.5 |
| PG 40:9 | [M+Cl]- | 851.4642 | 851.4635 | 0.8 |
| PI 36:1 | [M-H]- | 863.5647 | 863.5655 | -0.9 |
| PI O-34:4 | [M+Cl]- | 851.4865 | 851.4847 | 2.1 |
| PC O-40:7 | [M+Cl]- | 852.5641 | 852.5679 | -4.5 |
| PI O-34:3 | [M+Cl]- | 853.5017 | 853.5003 | 1.6 |
| PI 36:5 | [M-H]- | 855.5055 | 855.5029 | 3.0 |
| PG 42:3 | [M-H]- | 855.6095 | 855.6121 | -3.0 |
| PS 42:9 | [M-H]- | 856.5165 | 856.5134 | 3.6 |
| PI 38:5 | [M-H]- | 883.5318 | 883.5342 | -2.7 |
| PC O-40:5 | [M+Cl]- | 856.6016 | 856.5993 | 2.7 |
| PI 36:4 | [M-H]- | 857.5149 | 857.5186 | -4.3 |
| PE 42:4 | [M+Cl]- | 858.5777 | 858.5785 | -0.9 |
| PI 40:4 | [M-H]- | 913.5771 | 913.5812 | -4.5 |
| PI 40:5 | [M-H]- | 911.5612 | 911.5655 | -4.7 |
| PG 40:5 | [M+Cl]- | 859.5291 | 859.5261 | 3.5 |
| PE 42:3 | [M+Cl]- | 860.5954 | 860.5942 | 1.4 |
| PI 36:2 | [M-H]- | 861.5468 | 861.5499 | -3.6 |
| PI 34:5 | [M+Cl]- | 863.4499 | 863.4483 | 1.9 |
| PI 34:4 | [M+Cl]- | 865.4615 | 865.4639 | -2.8 |
| PG 44:12 | [M-H]- | 865.5016 | 865.5025 | -1.0 |
| PG 40:2 | [M+Cl]- | 865.5696 | 865.5731 | -4.0 |
| PI 34:3 | [M+Cl]- | 867.4782 | 867.4796 | -1.6 |

| | | | | |
|---|---|---|---|---|
| PI O-34:3 | [M-H]- | 817.5258 | 817.5236 | 2.7 |
| Hex2Cer 32:1;O2 | [M+Cl]- | 868.5517 | 868.5559 | -4.8 |
| PC 40:5 | [M+Cl]- | 870.5807 | 870.5785 | 2.5 |
| PI 34:1 | [M+Cl]- | 871.5073 | 871.5109 | -4.1 |
| PI O-36:3 | [M-H]- | 845.5551 | 845.5549 | 0.2 |
| PI O-36:4 | [M-H]- | 843.5380 | 843.5393 | -1.5 |
| PS 40:5 | [M+Cl]- | 872.5185 | 872.5214 | -3.3 |
| PI O-38:4 | [M-H]- | 871.5678 | 871.5706 | -3.2 |
| PI 38:6 | [M-H]- | 881.5151 | 881.5186 | -4.0 |
| PI O-38:5 | [M-H]- | 869.5509 | 869.5549 | -4.6 |
| PC O-42:5 | [M+Cl]- | 884.6267 | 884.6306 | -4.4 |
| PI O-38:6 | [M-H]- | 867.5377 | 867.5393 | -1.8 |
| PI 38:4 | [M-H]- | 885.5465 | 885.5499 | -3.8 |
| PC 44:0 | [M-CH3]- | 886.7296 | 886.7270 | 2.9 |
| PS 34:1 | [M-H]- | 760.5129 | 760.5134 | -0.7 |
| PG 42:5 | [M+Cl]- | 887.5555 | 887.5574 | -2.1 |
| PS 36:1 | [M-H]- | 788.5435 | 788.5447 | -1.5 |
| PG 44:1 | [M-H]- | 887.6724 | 887.6747 | -2.6 |
| PS 42:11 | [M+Cl]- | 888.4621 | 888.4588 | 3.7 |
| PS 44:6 | [M-H]- | 890.5873 | 890.5917 | -4.9 |
| PS 42:9 | [M+Cl]- | 892.4896 | 892.4901 | -0.6 |
| PS 42:8 | [M+Cl]- | 894.5028 | 894.5057 | -3.2 |
| PS 40:4 | [M-H]- | 838.5563 | 838.5604 | -4.9 |
| PC 42:7 | [M+Cl]- | 894.5793 | 894.5785 | 0.9 |
| Hex2Cer 34:1;O2 | [M+Cl]- | 896.5831 | 896.5872 | -4.6 |
| PS 42:6 | [M+Cl]- | 898.5374 | 898.5370 | 0.4 |
| PI 40:10 | [M-H]- | 901.4838 | 901.4873 | -3.9 |
| PI O-38:5 | [M+Cl]- | 905.5309 | 905.5316 | -0.8 |
| PI O-38:4 | [M+Cl]- | 907.5467 | 907.5473 | -0.7 |
| PI 40:6 | [M-H]- | 909.5468 | 909.5499 | -3.4 |

| | | | | |
|---|---|---|---|---|
| PG 44:7 | [M+Cl]$^-$ | 911.5612 | 911.5574 | 4.2 |
| PI 38:8 | [M+Cl]$^-$ | 913.4601 | 913.4639 | -4.2 |
| PG 44:6 | [M+Cl]$^-$ | 913.5749 | 913.5731 | 2.0 |
| PS 44:10 | [M+Cl]$^-$ | 918.5097 | 918.5057 | 4.4 |
| PI 38:4 | [M+Cl]$^-$ | 921.5225 | 921.5265 | -4.3 |
| PI 38:3 | [M+Cl]$^-$ | 923.5418 | 923.5422 | -0.4 |
| PI O-40:7 | [M+Cl]$^-$ | 929.5340 | 929.5316 | 2.6 |
| PS 44:3 | [M+Cl]$^-$ | 932.6198 | 932.6153 | 4.8 |
| PS O-40:7 | [M-H]$^-$ | 818.5305 | 818.5342 | -4.5 |
| PS O-42:7 | [M-H]$^-$ | 846.5625 | 846.5655 | -3.5 |
| PI 42:7 | [M-H]$^-$ | 935.5643 | 935.5655 | -1.3 |
| Hex2Cer 38:1;O2 | [M+Cl]$^-$ | 952.6488 | 952.6498 | -1.0 |
| PI 44:6 | [M-H]$^-$ | 965.6161 | 965.6125 | 3.7 |
| PI 42:6 | [M+Cl]$^-$ | 973.5533 | 973.5578 | -4.6 |
| PI 42:5 | [M+Cl]$^-$ | 975.5733 | 975.5735 | -0.2 |
| PI 44:12 | [M+Cl]$^-$ | 989.4961 | 989.4952 | 0.9 |
| PI 44:6 | [M+Cl]$^-$ | 1001.5884 | 1001.5891 | -0.7 |
| CaCl2 | [M+Cl]$^-$ | 144.8692 | 144.8697 | -3.5 |
| FeCl2 | [M+Cl]$^-$ | 160.8418 | 160.8421 | -1.9 |
| FeCl3 | [M+Cl]$^-$ | 197.8079 | 197.8080 | -0.5 |
| H2SO4 | [M-H]$^-$ | 96.9598 | 96.9601 | -3.1 |
| KCl | [M+Cl]$^-$ | 108.9018 | 108.9020 | -1.8 |
| MgCl2 | [M+Cl]$^-$ | 130.8892 | 130.8893 | -0.8 |
| NaCl | [M+Cl]$^-$ | 92.9277 | 92.9280 | -3.2 |
| ZnCl2 | [M+Cl]$^-$ | 170.8330 | 170.8332 | -1.2 |

The Relative mass error is calculated by :

$$relative\ error = \frac{experiment\ m/z - theroretical\ m/z}{theroretical\ m/z} \times 10^6$$

**Table S2. Molecular assignments within five ppm relative error in positive ion mode.**

| Assignment | Adduct form | Experimental *m/z* | Theoretical *m/z* | Relative error (ppm) |
|---|---|---|---|---|
| CAR 10:1;O2 | $[M+Na]^+$ | 368.2027 | 368.2044 | -4.6 |
| CAR 18:0 | $[M+H]^+$ | 428.3748 | 428.3734 | 3.3 |
| CAR 18:1 | $[M+H]^+$ | 426.3597 | 426.3578 | 4.5 |
| CAR 20:0;O | $[M+K]^+$ | 510.3572 | 510.3555 | 3.3 |
| CAR 22:0 | $[M+K]^+$ | 522.3907 | 522.3919 | -2.3 |
| CAR 22:5 | $[M+Na]^+$ | 496.3423 | 496.3397 | 5.2 |
| CAR 22:6 | $[M+Na]^+$ | 494.3251 | 494.3241 | 2.0 |
| CE 20:1 | $[M+K]^+$ | 717.5935 | 717.5946 | -1.5 |
| CE 20:2 | $[M+Na]^+$ | 699.6031 | 699.605 | -2.7 |
| CE 22:1 | $[M+K]^+$ | 745.6253 | 745.6259 | -0.8 |
| Cer 34:0;O | $[M+K]^+$ | 562.4956 | 562.496 | -0.7 |
| Cer 34:1;O3 | $[M+H]^+$ | 554.5161 | 554.5143 | 3.2 |
| Cer 36:1;O2 | $[M+K]^+$ | 604.5055 | 604.5065 | -1.7 |
| Cer 40:0;O | $[M+K]^+$ | 646.5905 | 646.5899 | 0.9 |
| Cer 40:0;O2 | $[M+K]^+$ | 662.5829 | 662.5848 | -2.9 |
| Cer 42:0;O | $[M+K]^+$ | 674.6191 | 674.6212 | -3.1 |
| Cer 42:2;O | $[M+H]^+$ | 632.6348 | 632.634 | 1.3 |
| Cer 44:0;O | $[M+K]^+$ | 702.6495 | 702.6525 | -4.3 |
| CerP 40:1;O2 | $[M+Na]^+$ | 724.559 | 724.5615 | -3.5 |
| CerP 44:2;O2 | $[M+H]^+$ | 756.6291 | 756.6265 | 3.4 |
| CerPE 34:2;O2 | $[M+Na]^+$ | 681.4911 | 681.4942 | -4.5 |

| | | | | |
|---|---|---|---|---|
| CerPE 34:3;O2 | $[M+Na]^+$ | 679.4769 | 679.4785 | -2.4 |
| CerPE 36:1;O2 | $[M+Na]^+$ | 711.5411 | 711.5411 | 0.0 |
| CerPE 40:1;O2 | $[M+H]^+$ | 745.6253 | 745.6218 | 4.7 |
| CerPE 40:1;O2 | $[M+K]^+$ | 783.5741 | 783.5777 | -4.6 |
| DG 32:0 | $[M+H]^+$ | 569.5111 | 569.5139 | -4.9 |
| DG 32:0 | $[M+Na]^+$ | 591.495 | 591.4959 | -1.5 |
| DG 32:1 | $[M+H]^+$ | 567.4958 | 567.4983 | -4.4 |
| DG 34:1 | $[M+Na]^+$ | 617.5119 | 617.5115 | 0.6 |
| DG 36:2 | $[M+Na]^+$ | 643.5297 | 643.5272 | 3.9 |
| DG 38:0 | $[M+H]^+$ | 653.6067 | 653.6078 | -1.7 |
| DG 38:4 | $[M+Na]^+$ | 667.5276 | 667.5272 | 0.6 |
| DG 38:6 | $[M+Na]^+$ | 663.4979 | 663.4959 | 3.0 |
| DG 38:7 | $[M+K]^+$ | 677.4551 | 677.4542 | 1.3 |
| DG 42:10 | $[M+Na]^+$ | 711.4949 | 711.4959 | -1.4 |
| Hex2Cer 34:1;O2 | $[M+Na]^+$ | 884.6111 | 884.607 | 4.6 |
| Hex2Cer 36:0;O2 | $[M+H]^+$ | 892.6755 | 892.672 | 3.9 |
| Hex2Cer 36:1;O2 | $[M+Na]^+$ | 912.6415 | 912.6383 | 3.5 |
| Hex2Cer 38:0;O2 | $[M+K]^+$ | 958.6615 | 958.6591 | 2.5 |
| Hex2Cer 38:1;O2 | $[M+Na]^+$ | 940.6707 | 940.6696 | 1.2 |
| Hex2Cer 40:0;O2 | $[M+K]^+$ | 986.6897 | 986.6904 | -0.7 |
| Hex2Cer 40:1;O2 | $[M+K]^+$ | 984.6715 | 984.6748 | -3.4 |
| HexCer 32:1;O2 | $[M+Na]^+$ | 694.5198 | 694.5228 | -4.3 |
| HexCer 36:2;O2 | $[M+H]^+$ | 726.5888 | 726.5878 | 1.4 |
| HexCer 38:0;O2 | $[M+K]^+$ | 796.6084 | 796.6063 | 2.6 |
| HexCer 40:0;O2 | $[M+K]^+$ | 824.641 | 824.6376 | 4.1 |

| | | | | |
|---|---|---|---|---|
| HexCer 40:2;O2 | $[M+H]^+$ | 782.652 | 782.6504 | 2.0 |
| HexCer 42:2;O2 | $[M+Na]^+$ | 832.6602 | 832.6637 | -4.2 |
| IPC 34:0;O3 | $[M+Na]^+$ | 820.5301 | 820.531 | -1.1 |
| LPE 22:0 | $[M+H]^+$ | 538.3874 | 538.3867 | 1.3 |
| LPE 22:1 | $[M+H]^+$ | 536.3695 | 536.3711 | -3.0 |
| LPE 22:5 | $[M+K]^+$ | 566.2642 | 566.2643 | -0.2 |
| LPE O-20:0 | $[M+Na]^+$ | 518.3579 | 518.3581 | -0.4 |
| LPE O-22:0;O | $[M+H]^+$ | 540.4034 | 540.4024 | 1.9 |
| LPI 18:0 | $[M+K]^+$ | 639.2917 | 639.2906 | 1.7 |
| LPI O-16:1 | $[M+K]^+$ | 595.2645 | 595.2644 | 0.2 |
| LPS 16:0 | $[M+K]^+$ | 536.2368 | 536.2385 | -3.2 |
| LPS 18:1 | $[M+K]^+$ | 562.2566 | 562.2542 | 4.3 |
| LPS 18:4 | $[M+Na]^+$ | 540.2318 | 540.2333 | -2.8 |
| LPS 20:2 | $[M+K]^+$ | 588.2713 | 588.2698 | 2.5 |
| LPS 22:0 | $[M+H]^+$ | 582.3786 | 582.3765 | 3.6 |
| LPS O-16:1 | $[M+K]^+$ | 520.2416 | 520.2436 | -3.8 |
| PA 34:1 | $[M+Na]^+$ | 697.4813 | 697.4779 | 4.9 |
| PA 36:1 | $[M+Na]^+$ | 725.5088 | 725.5092 | -0.6 |
| PA 36:1 | $[M+K]^+$ | 741.48 | 741.4831 | -4.2 |
| PA 36:2 | $[M+Na]^+$ | 723.4965 | 723.4935 | 4.1 |
| PA 38:2 | $[M+K]^+$ | 767.5017 | 767.4988 | 3.8 |
| PA 38:3 | $[M+K]^+$ | 765.4858 | 765.4831 | 3.5 |
| PA 38:5 | $[M+H]^+$ | 723.4965 | 723.4959 | 0.8 |
| PA 38:5 | $[M+K]^+$ | 761.4547 | 761.4518 | 3.8 |
| PA 40:5 | $[M+H]^+$ | 751.5297 | 751.5272 | 3.3 |

| | | | | |
|---|---|---|---|---|
| PA 40:6 | $[M+H]^+$ | 749.5145 | 749.5116 | 3.9 |
| PA 40:7 | $[M+K]^+$ | 785.455 | 785.4518 | 4.1 |
| PC 30:0 | $[M+H]^+$ | 706.5406 | 706.5381 | 3.5 |
| PC 30:2 | $[M+H]^+$ | 702.5072 | 702.5068 | 0.6 |
| PC 31:1 | $[M+H]^+$ | 718.5395 | 718.5381 | 1.9 |
| PC 32:0 | $[M+K]^+$ | 772.5279 | 772.5253 | 3.4 |
| PC 32:1 | $[M+K]^+$ | 770.5123 | 770.5097 | 3.4 |
| PC 34:0 | $[M+H]^+$ | 762.6015 | 762.6007 | 1.0 |
| PC 34:0 | $[M+Na]^+$ | 784.5817 | 784.5827 | -1.3 |
| PC 34:0 | $[M+K]^+$ | 800.5533 | 800.5566 | -4.1 |
| PC 34:1 | $[M+K]^+$ | 798.5445 | 798.541 | 4.4 |
| PC 34:2 | $[M+K]^+$ | 796.5287 | 796.5253 | 4.3 |
| PC 34:3 | $[M+H]^+$ | 756.5553 | 756.5538 | 2.0 |
| PC 34:4 | $[M+H]^+$ | 754.5399 | 754.5381 | 2.4 |
| PC 36:0 | $[M+H]^+$ | 790.6285 | 790.632 | -4.4 |
| PC 36:1 | $[M+H]^+$ | 788.6189 | 788.6164 | 3.2 |
| PC 36:1 | $[M+Na]^+$ | 810.6008 | 810.5983 | 3.1 |
| PC 36:1 | $[M+K]^+$ | 826.575 | 826.5723 | 3.3 |
| PC 36:2 | $[M+K]^+$ | 824.5606 | 824.5566 | 4.9 |
| PC 36:3 | $[M+K]^+$ | 822.5408 | 822.541 | -0.2 |
| PC 36:4 | $[M+H]^+$ | 782.5698 | 782.5694 | 0.5 |
| PC 36:7 | $[M+K]^+$ | 814.4746 | 814.4784 | -4.7 |
| PC 38:4 | $[M+K]^+$ | 848.5599 | 848.5566 | 3.9 |
| PC 38:5 | $[M+H]^+$ | 808.5869 | 808.5851 | 2.2 |
| PC 38:5 | $[M+K]^+$ | 846.5406 | 846.541 | -0.5 |

| | | | | |
|---|---|---|---|---|
| PC 38:6 | [M+H]+ | 806.572 | 806.5694 | 3.2 |
| PC 40:1 | [M+H]+ | 844.6781 | 844.679 | -1.1 |
| PC 40:4 | [M+Na]+ | 860.6136 | 860.614 | -0.5 |
| PC 40:7 | [M+H]+ | 832.5873 | 832.5851 | 2.6 |
| PC 42:1 | [M+H]+ | 872.7098 | 872.7103 | -0.6 |
| PC 42:1 | [M+Na]+ | 894.6952 | 894.6922 | 3.4 |
| PC 42:1 | [M+K]+ | 910.6677 | 910.6662 | 1.6 |
| PC 42:2 | [M+Na]+ | 892.6755 | 892.6766 | -1.2 |
| PC 42:6 | [M+Na]+ | 884.6111 | 884.614 | -3.3 |
| PC 44:6 | [M+Na]+ | 912.6415 | 912.6453 | -4.2 |
| PC O-30:1 | [M+Na]+ | 712.5219 | 712.5252 | -4.6 |
| PC O-36:0 | [M+Na]+ | 798.6323 | 798.6347 | -3.0 |
| PC O-36:4 | [M+K]+ | 806.5458 | 806.546 | -0.2 |
| PC O-38:3 | [M+Na]+ | 820.6225 | 820.6191 | 4.1 |
| PC O-40:3 | [M+Na]+ | 848.6532 | 848.6504 | 3.3 |
| PC O-40:4 | [M+Na]+ | 846.6337 | 846.6347 | -1.2 |
| PE 30:2 | [M+H]+ | 660.4611 | 660.4599 | 1.8 |
| PE 34:1 | [M+H]+ | 718.5395 | 718.5381 | 1.9 |
| PE 38:3 | [M+H]+ | 770.5701 | 770.5694 | 0.9 |
| PE 40:1 | [M+H]+ | 802.6315 | 802.632 | -0.6 |
| PE 42:2 | [M+H]+ | 828.6461 | 828.6477 | -1.9 |
| PE 42:2 | [M+K]+ | 866.6049 | 866.6036 | 1.5 |
| PE 44:2 | [M+K]+ | 894.6371 | 894.6349 | 2.5 |
| PE O-36:1 | [M+H]+ | 732.5878 | 732.5902 | -3.3 |
| PE O-38:2 | [M+Na]+ | 780.5885 | 780.5878 | 0.9 |

| | | | | |
|---|---|---|---|---|
| PE O-38:6 | $[M+Na]^+$ | 772.5279 | 772.5252 | 3.5 |
| PE O-42:2 | $[M+K]^+$ | 852.6214 | 852.6243 | -3.4 |
| PG 34:2 | $[M+Na]^+$ | 769.4998 | 769.499 | 1.0 |
| PG 34:5 | $[M+K]^+$ | 779.4225 | 779.426 | -4.5 |
| PG 36:0 | $[M+Na]^+$ | 801.5587 | 801.5616 | -3.6 |
| PG 36:1 | $[M+Na]^+$ | 799.5473 | 799.546 | 1.6 |
| PG 38:1 | $[M+Na]^+$ | 827.5799 | 827.5773 | 3.1 |
| PG 38:2 | $[M+Na]^+$ | 825.5651 | 825.5616 | 4.2 |
| PG 38:4 | $[M+Na]^+$ | 821.5339 | 821.5303 | 4.4 |
| PG 38:5 | $[M+K]^+$ | 835.4877 | 835.4886 | -1.1 |
| PG 40:0 | $[M+K]^+$ | 873.6004 | 873.5981 | 2.6 |
| PG 40:3 | $[M+H]^+$ | 829.5975 | 829.5953 | 2.7 |
| PG 40:4 | $[M+Na]^+$ | 849.5639 | 849.5616 | 2.7 |
| PG 40:6 | $[M+Na]^+$ | 845.5338 | 845.5303 | 4.1 |
| PG 40:7 | $[M+H]^+$ | 821.5339 | 821.5327 | 1.5 |
| PG 42:2 | $[M+Na]^+$ | 881.6262 | 881.6242 | 2.3 |
| PG 42:6 | $[M+Na]^+$ | 873.5655 | 873.5616 | 4.5 |
| PG 44:1 | $[M+Na]^+$ | 911.6746 | 911.6712 | 3.7 |
| PG dO-40:0 | $[M+K]^+$ | 845.6437 | 845.6396 | 4.8 |
| PG O-42:2 | $[M+K]^+$ | 883.6231 | 883.6189 | 4.8 |
| PI 28:0 | $[M+K]^+$ | 793.4263 | 793.4264 | -0.1 |
| PI 28:1 | $[M+K]^+$ | 791.4096 | 791.4107 | -1.4 |
| PI 30:4;O2 | $[M+K]^+$ | 845.3816 | 845.3849 | -3.9 |
| PI 34:4 | $[M+Na]^+$ | 853.4852 | 853.4837 | 1.8 |
| PI 34:6 | $[M+K]^+$ | 865.4228 | 865.4264 | -4.2 |

| | | | | |
|---|---|---|---|---|
| PI 36:1 | $[M+H]^+$ | 865.5785 | 865.5801 | -1.8 |
| PI 36:8 | $[M+K]^+$ | 889.4254 | 889.4264 | -1.1 |
| PI 38:2 | $[M+Na]^+$ | 913.5797 | 913.5776 | 2.3 |
| PI 40:10 | $[M+K]^+$ | 941.4565 | 941.4577 | -1.3 |
| PI 40:5 | $[M+H]^+$ | 913.5797 | 913.5801 | -0.4 |
| PI 42:11 | $[M+K]^+$ | 967.4735 | 967.4733 | 0.2 |
| PI 42:5 | $[M+Na]^+$ | 963.5921 | 963.5933 | -1.2 |
| PI 44:8 | $[M+H]^+$ | 963.5921 | 963.5957 | -3.7 |
| PI O-34:4 | $[M+K]^+$ | 855.4825 | 855.4784 | 4.8 |
| PS 34:6 | $[M+K]^+$ | 790.4052 | 790.4056 | -0.5 |
| PS 36:6 | $[M+K]^+$ | 818.4343 | 818.4369 | -3.2 |
| SM 30:0;O2 | $[M+H]^+$ | 649.5295 | 649.5279 | 2.5 |
| SM 30:0;O2 | $[M+Na]^+$ | 671.5079 | 671.5098 | -2.8 |
| SM 34:0;O2 | $[M+H]^+$ | 705.5892 | 705.5905 | -1.8 |
| SM 36:0;O2 | $[M+Na]^+$ | 755.6008 | 755.6037 | -3.8 |
| SM 36:1;O2 | $[M+Na]^+$ | 753.5917 | 753.5881 | 4.8 |
| SM 36:1;O2 | $[M+K]^+$ | 769.5652 | 769.562 | 4.2 |
| SM 36:2;O2 | $[M+H]^+$ | 729.594 | 729.5905 | 4.8 |
| SM 38:0;O2 | $[M+H]^+$ | 761.6508 | 761.6531 | -3.0 |
| SM 38:1;O2 | $[M+H]^+$ | 759.6408 | 759.6374 | 4.5 |
| SM 38:2;O2 | $[M+H]^+$ | 757.6209 | 757.6218 | -1.2 |
| SM 40:1;O2 | $[M+H]^+$ | 787.6711 | 787.6687 | 3.0 |
| SM 40:1;O2 | $[M+Na]^+$ | 809.6468 | 809.6507 | -4.8 |
| SM 42:1;O2 | $[M+H]^+$ | 815.7012 | 815.7 | 1.5 |
| TG 40:1 | $[M+Na]^+$ | 715.5834 | 715.5847 | -1.8 |

| | | | | |
|---|---|---|---|---|
| TG 44:2 | $[M+Na]^+$ | 769.6306 | 769.6317 | -1.4 |
| TG 46:0 | $[M+Na]^+$ | 801.6909 | 801.6943 | -4.2 |
| TG 48:0 | $[M+Na]^+$ | 829.7219 | 829.7256 | -4.5 |
| TG 48:1 | $[M+Na]^+$ | 827.7082 | 827.7099 | -2.1 |
| TG 50:1 | $[M+Na]^+$ | 855.7413 | 855.7412 | 0.1 |
| TG 50:2 | $[M+Na]^+$ | 853.7284 | 853.7256 | 3.3 |
| TG 50:2 | $[M+K]^+$ | 869.702 | 869.6995 | 2.9 |
| TG 52:1 | $[M+Na]^+$ | 883.7689 | 883.7725 | -4.1 |
| TG 52:2 | $[M+Na]^+$ | 881.7584 | 881.7569 | 1.7 |
| TG 52:2 | $[M+K]^+$ | 897.7311 | 897.7308 | 0.3 |
| TG 52:3 | $[M+Na]^+$ | 879.7437 | 879.7412 | 2.8 |
| TG 52:4 | $[M+Na]^+$ | 877.7292 | 877.7256 | 4.1 |
| TG 52:5 | $[M+Na]^+$ | 875.7106 | 875.7099 | 0.8 |
| TG 52:5 | $[M+K]^+$ | 891.6852 | 891.6838 | 1.6 |
| TG 52:9 | $[M+K]^+$ | 883.6231 | 883.6212 | 2.2 |
| TG 54:2 | $[M+Na]^+$ | 909.7848 | 909.7882 | -3.7 |
| TG 54:2 | $[M+K]^+$ | 925.7605 | 925.7621 | -1.7 |
| TG 54:3 | $[M+Na]^+$ | 907.7741 | 907.7725 | 1.8 |
| TG 54:3 | $[M+K]^+$ | 923.7469 | 923.7464 | 0.5 |
| TG 54:4 | $[M+Na]^+$ | 905.7589 | 905.7569 | 2.2 |
| TG 54:4 | $[M+K]^+$ | 921.733 | 921.7308 | 2.4 |
| TG 54:5 | $[M+Na]^+$ | 903.7435 | 903.7412 | 2.5 |
| TG 54:5 | $[M+K]^+$ | 919.7166 | 919.7151 | 1.6 |
| TG 54:6 | $[M+Na]^+$ | 901.7282 | 901.7256 | 2.9 |
| TG 54:6 | $[M+K]^+$ | 917.7001 | 917.6995 | 0.7 |

| | | | | |
|---|---|---|---|---|
| TG 56:3 | $[M+Na]^+$ | 935.8036 | 935.8038 | -0.2 |
| TG 56:4 | $[M+Na]^+$ | 933.7891 | 933.7882 | 1.0 |

The Relative mass error is calculated by :

$$relative\ error\ = \frac{experiment\ \ m/z\ - theroretical\ \ m/z}{theroretical\ m/z} \times\ 10^6$$

**Table S3. Molecular identification by tandem mass spectrometry**

| Identification | Fragments | Experimental *m/z* | Relative error (ppm) | Collison energy eV |
|---|---|---|---|---|
| GalNAcS | 58;97;242;201;198 | 300.0386 | -2.9 | 20 |
| PA 14:1/18:0 | 283;419 | 645.4485 | -2.5 | 25 |
| PE 14:1/18:0 | 283;480 | 688.4921 | -0.3 | 20 |
| PA 36:2 | 283;279;415;419;437 | 699.4949 | -3.0 | 25 |
| PE 16:0/18:2 | 279;255 | 714.5059 | -2.8 | 25 |
| PE 16:0/18:1 | 281;255 | 716.5217 | -2.7 | 10 |
| PE 16:0/18:0 | 255;283 | 718.5379 | -1.8 | 10 |
| PE 18:0/18:2 | 279;283;480 | 742.5374 | -2.4 | 25 |
| PG 18:0/18:2 | 279;283;511 | 773.5302 | -4.7 | 25 |
| PS 18:0/18:1 | 283;281;419;437 | 788.5422 | -3.2 | 25 |
| PI 16:0/18:1 | 281;255;417;579 | 835.5317 | -3.0 | 30 |
| PI 16:0/18:0 | 283;255;419;437 | 837.5475 | -2.9 | 25 |
| PS 18:0/18:1 | 255;281;673 | 760.5113 | -2.8 | 25 |
| LPE 18:1 | 281 | 478.2929 | -2.1 | 30 |

**Table S4. Signal detected in DHB and NEDC in positive and negative ion mode, respectively.**

| m/z | Polarity | Matrix | Normalized Intensity (%) |
|---|---|---|---|
| 109.0281 | Positive | DHB | 2.5 |
| 110.0366 | Positive | DHB | 1.6 |
| 136.0156 | Positive | DHB | 34.7 |
| 137.0227 | Positive | DHB | 100 |
| 138.0279 | Positive | DHB | 11.6 |
| 139.0387 | Positive | DHB | 1.3 |
| 154.0257 | Positive | DHB | 24 |
| 155.0335 | Positive | DHB | 20.7 |
| 156.0402 | Positive | DHB | 3 |
| 257.0436 | Positive | DHB | 1.5 |
| 273.0398 | Positive | DHB | 4.9 |
| 289.0584 | Positive | DHB | 8.2 |
| 290.0657 | Positive | DHB | 2.1 |
| 330.3366 | Positive | DHB | 1.3 |
| 387.0727 | Positive | DHB | 1.2 |
| 34.9661 | Negative | NEDC | 7.8 |
| 36.9632 | Negative | NEDC | 2.4 |
| 96.9587 | Negative | NEDC | 100 |
| 98.9547 | Negative | NEDC | 5.2 |
| 132.8668 | Negative | NEDC | 1.7 |
| 134.8648 | Negative | NEDC | 2.3 |

| | | | |
|---|---|---|---|
| 144.8684 | Negative | NEDC | 1.9 |
| 146.8667 | Negative | NEDC | 2.2 |
| 158.0377 | Negative | NEDC | 1.1 |
| 178.8383 | Negative | NEDC | 1.1 |
| 185.1106 | Negative | NEDC | 1.1 |
| 194.9288 | Negative | NEDC | 4 |
| 195.8101 | Negative | NEDC | 1.3 |
| 252.7815 | Negative | NEDC | 1.9 |
| 254.7805 | Negative | NEDC | 31.7 |
| 256.7782 | Negative | NEDC | 35.6 |
| 258.7746 | Negative | NEDC | 15.7 |
| `260.7722 | Negative | NEDC | 3.6 |
| 265.0652 | Negative | NEDC | 1.9 |
| 297.1549 | Negative | NEDC | 1.5 |
| 311.1704 | Negative | NEDC | 2.1 |
| 325.1856 | Negative | NEDC | 1.9 |
| 342.1368 | Negative | NEDC | 1.2 |
| 343.1475 | Negative | NEDC | 1.2 |
| 351.1649 | Negative | NEDC | 1.7 |

* Only signals with intensity higher than 1% of the base peak were tabulated.

**Table S5. The number of technical replicates performed to evaluate metabolome change at knee growth plate in different age.**

| | |
|---|---|
| Species | SD Rat |
| Age | 4 weeks old  and 12 weeks old |
| Sex | Male |
| Number of animal | One for each age |
| Number of technical replicates | 3 |

**Table S6. Relative mass error of all detected isotopic peaks, experimental isotopic distribution measured on tissue, metal chloride standards, and theoretical isotopic ratio.**

| Metal Ions | Isotopologues | Experimental m/z | True m/z | Relative mass error(ppm) | Isotopic ratio | Calculated Isotopic Ratio | Isotopic Ratio (Standard) |
|---|---|---|---|---|---|---|---|
| $CaCl_3^-$ | M0 | 144.8692 | 144.8697 | -3.4 | 100.0 | 100.0 | 100.0 |
| | M1 | 146.8661 | 146.8667 | -4.4 | 82.5 | 96.7 | 98.3 |
| | M2 | 148.8637 | 148.8637 | 0.0 | 19.3 | 33.5 | 35.3 |
| $FeCl_3^-$ | M0 | 158.8462 | 158.8467 | -3.2 | 9.4 | 6.0 | 5.5 |
| | M1 | 160.8418 | 160.8421 | -2.1 | 76.3 | 100.0 | 100.0 |
| | M2 | 161.8421 | 161.8425 | -2.5 | 1.5 | 2.2 | -2.1 |
| | M3 | 162.8389 | 162.8391 | -1.4 | 100.0 | 92.6 | 84.2 |
| | M4 | 163.8393 | 163.8395 | -1.5 | 1.6 | 2.1 | 3.0 |
| | M5 | 164.8360 | 164.8362 | -1.0 | 38.0 | 29.4 | 26.3 |
| $FeCl_4^-$ | M0 | 193.8154 | 193.8156 | -0.9 | 11.4 | 4.8 | 5.4 |

| | | | | | | | |
|---|---|---|---|---|---|---|---|
| | M1 | 195.8103 | 195.8110 | -3.7 | 100.0 | 81.8 | 91.7 |
| | M2 | 196.8105 | 196.8114 | -4.3 | 0.4 | 1.7 | 2.9 |
| | M3 | 197.8079 | 197.8080 | -0.5 | 86.5 | 100.0 | 100.0 |
| | M5 | 199.8049 | 199.8050 | -0.6 | 38.6 | 47.4 | 50.0 |
| | M7 | 201.8018 | 201.8021 | -1.3 | 5.7 | 10.1 | 14.8 |
| $ZnCl_3^-$ | M0 | 168.8357 | 168.8362 | -3.3 | 66.4 | 65.6 | 51.6 |
| | M1 | 170.8330 | 170.8332 | -1.4 | 100.0 | 100.0 | 100.0 |
| | M3 | 172.8305 | 172.8308 | -1.6 | 79.1 | 80.3 | 73.5 |
| | M5 | 174.8278 | 174.8285 | -3.8 | 35.9 | 38.0 | 53.7 |
| $MgCl_3^-$ | M0 | 128.8916 | 128.8921 | -4.3 | 98.6 | 91.0 | 91.2 |
| | M1 | 129.8924 | 129.8929 | -4.2 | 6.3 | 11.5 | 17.5 |
| | M2 | 130.8892 | 130.8893 | -0.5 | 100.0 | 100.0 | 100.0 |
| | M3 | 131.8898 | 131.8900 | -1.5 | 6.3 | 11.1 | 10.7 |
| | M4 | 132.8860 | 132.8864 | -3.0 | 41.6 | 40.1 | 41.5 |
| $NaCl_2^-$ | M0 | 92.9277 | 92.9280 | -3.7 | 100.0 | 100.0 | 100.0 |
| | M1 | 94.9250 | 94.9251 | -1.3 | 66.5 | 64.0 | 73.2 |

| | | | | | | | |
|---|---|---|---|---|---|---|---|
| $KCl_2^-$ | M0 | 108.9018 | 108.9020 | -1.5 | 100.0 | 100.0 | 100.0 |
| | M1 | 110.8986 | 110.8991 | -4.7 | 56.8 | 71.2 | 63.2 |

121

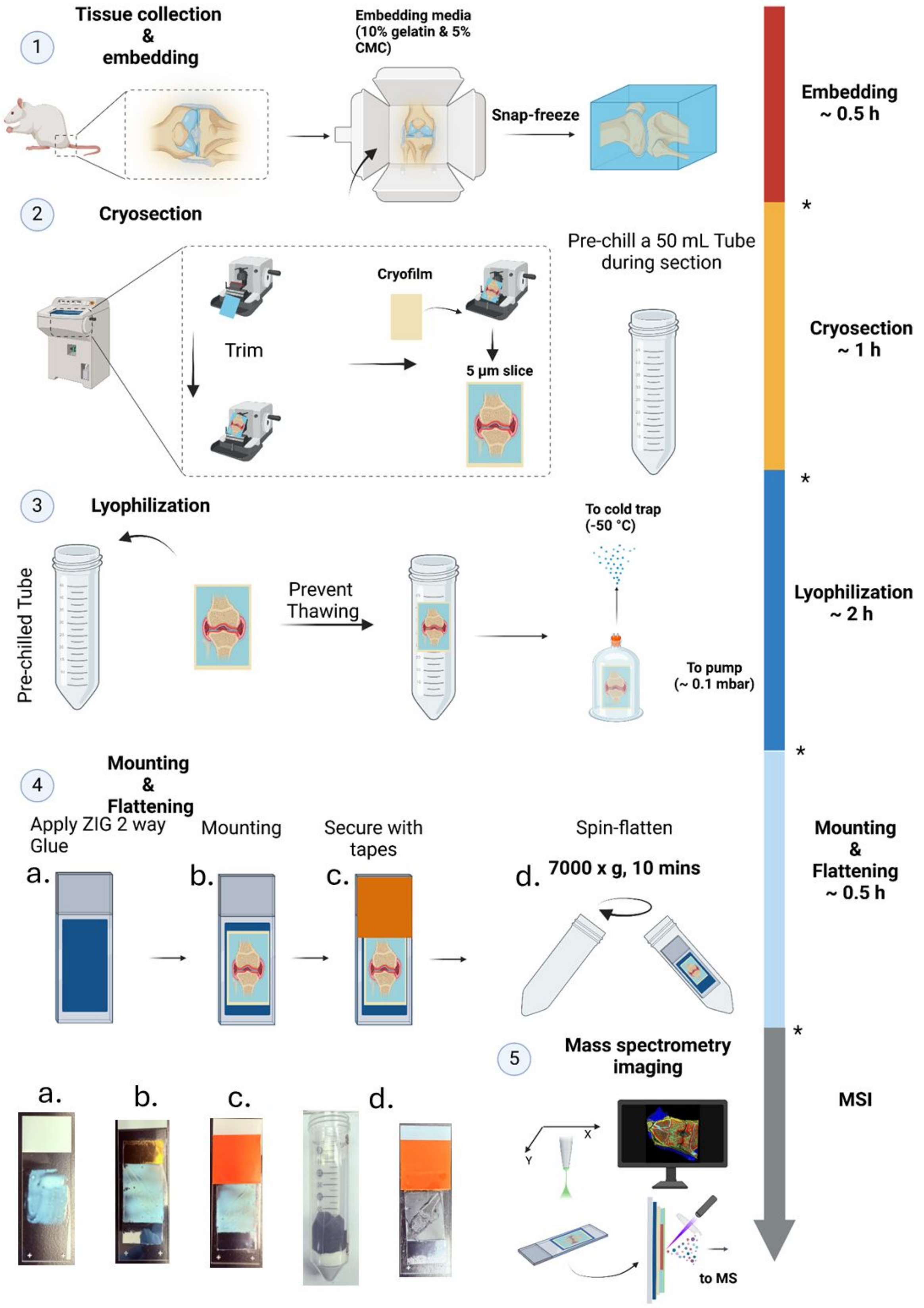

1 Tissue collection & embedding
Embedding media (10% gelatin & 5% CMC)
Snap-freeze
2 Cryosection
Pre-chill a 50 mL Tube during section
Cryofilm
Trim
5 µm slice
3 Lyophilization
Pre-chilled Tube
Prevent Thawing
To cold trap (-50 °C)
To pump (~ 0.1 mbar)
4 Mounting & Flattening
Apply ZIG 2 way Glue
Mounting
Secure with tapes
Spin-flatten
7000 x g, 10 mins
a.
b.
c.
d.
5 Mass spectrometry imaging
X
Y
to MS
Embedding ~ 0.5 h
Cryosection ~ 1 h
Lyophilization ~ 2 h
Mounting & Flattening ~ 0.5 h
MSI

**Figure S1. Detailed procedure of the proposed method.** The workflow presented in this work contains five steps: tissue collection/embedding, cryosectioning, lyophilization, mounting and flattening, and mass spectrometry measurement. As a critical step in our workflow, stage 4 was marked with (a), (b), (c), and (d) in the lower-left corner; the corresponding real pictures were provided for better illustration. The entire process can be finished within a day. However, it can be paused at any stage indicated with an asterisk (*). *Created in BioRender. Wang, J. (2025) https://BioRender.com/b78p083*

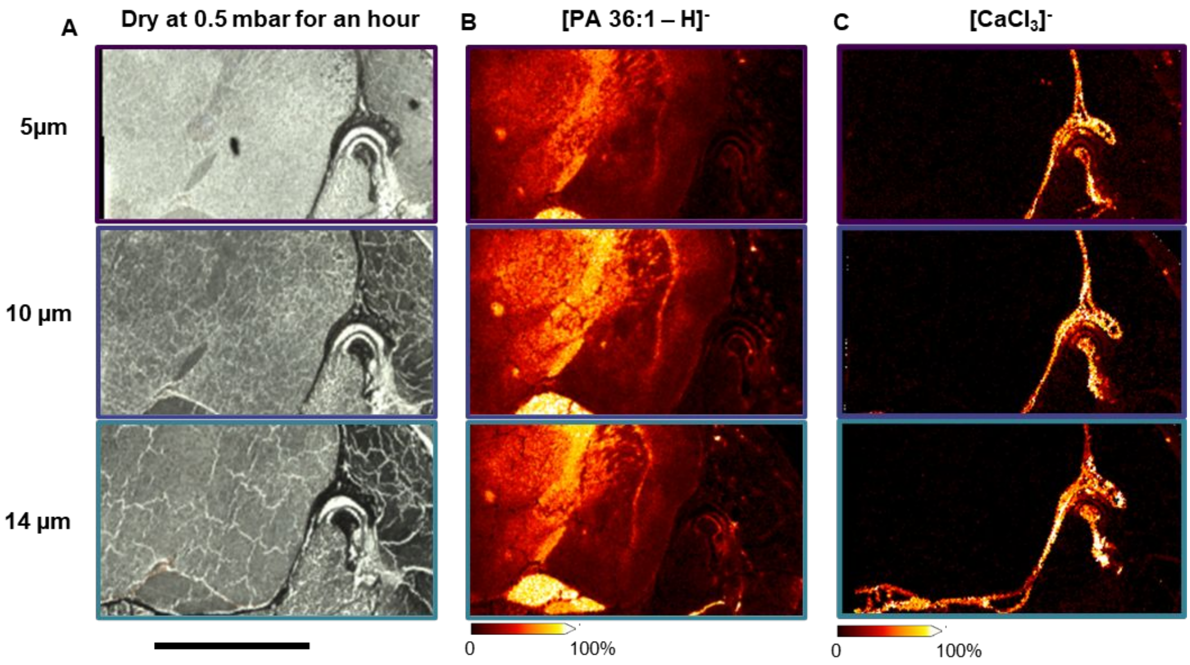


**Figure S2. Fresh frozen mouse skull sectioned at different thicknesses and dried by lyophilization.** Bright field scan of mouse serial section with 5, 10, and 14 µm thickness (A). Ion image of PA 36:1 of mouse serial section with 5, 10, and 14 µm thickness (B). Ion images of $CaCl_3^-$ of mouse serial section with 5, 10, and 14 µm thickness (C). The scale at the lower left corner represents 3 mm.

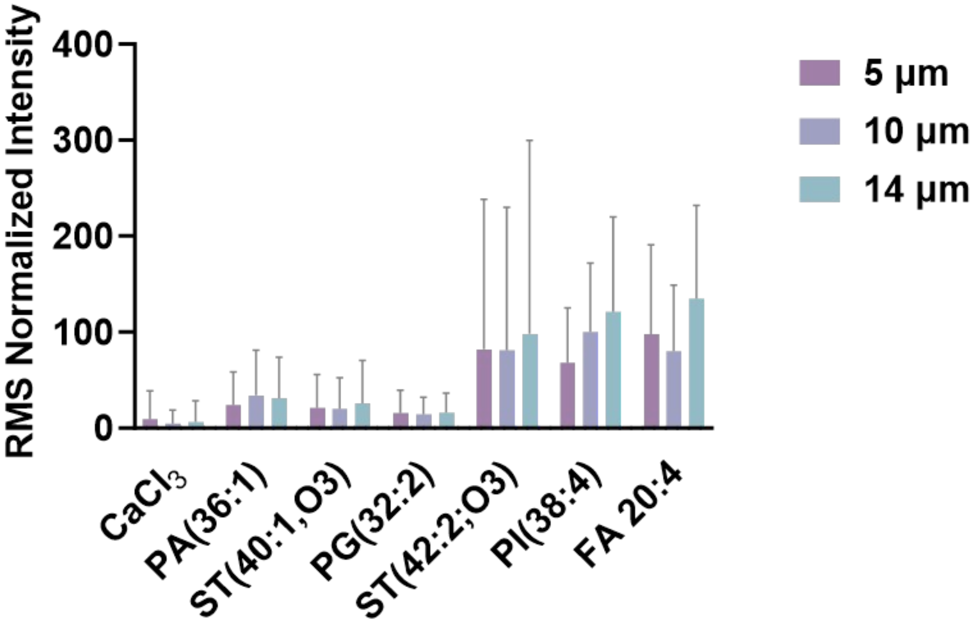


**Figure S3. The average intensity of fresh frozen mouse skull sectioned at different thicknesses and dried by lyophilization.** Root mean square normalized intensities of $CaCl_3^-$, PA (36:1), ST (40:1; O3), PG (32:2), ST (42:2; O3), PI (38:4) and FA 20:4 were shown.

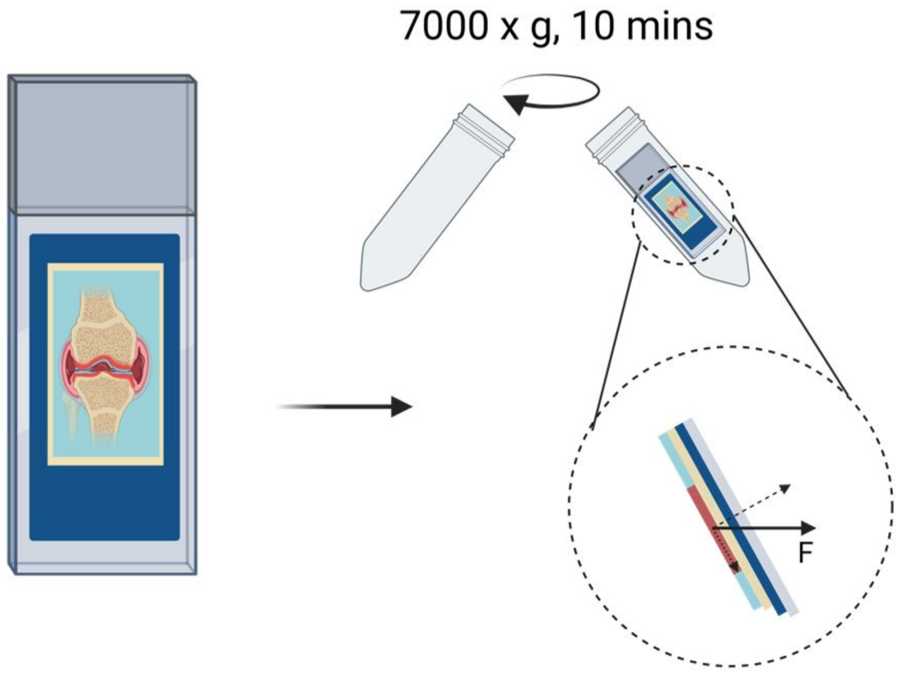


**Figure S4. Schematic diagram of centrifuge mounting.**

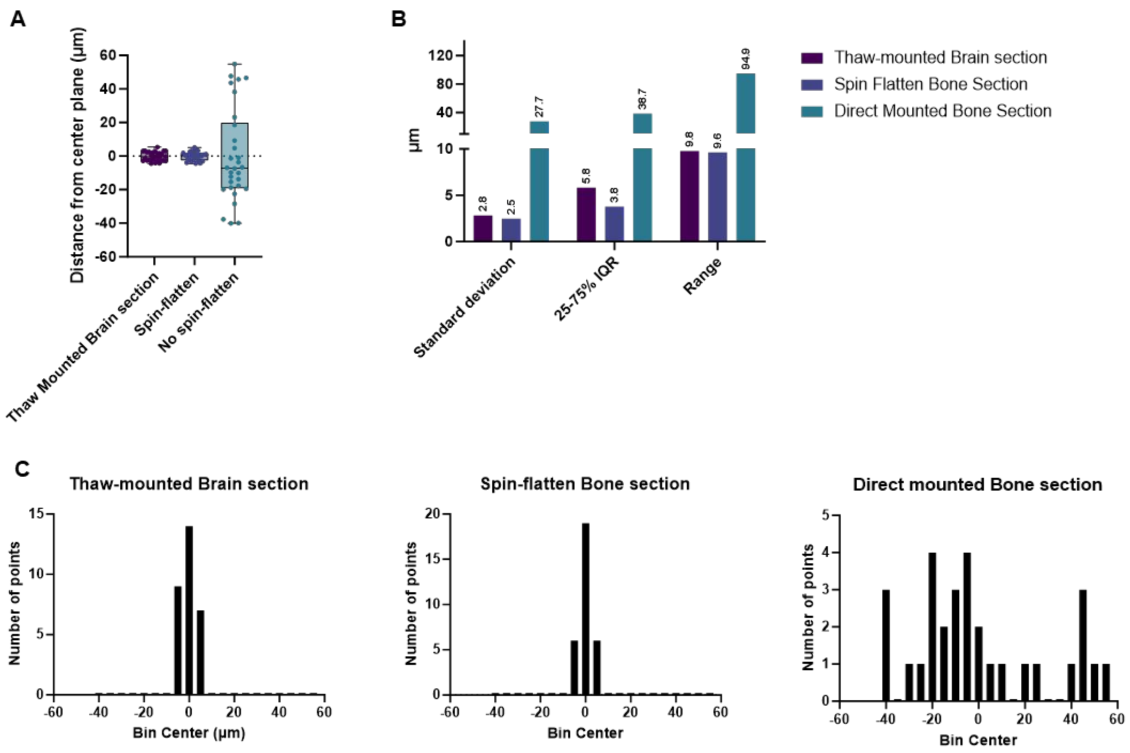


**Figure S5. The surface height of random points on tissue was determined by confocal microscopy with a 20X objective and 56 µm pinhole.** (A) The relative variation of randomly selected points was shown (since the surface level is different, the average of surface height was assumed to be the center plane, and deviation from the center plane is considered as non-flatness) of the thaw-mounted brain section, spin-flattened bone section, and bone section without spin-flattened. (B) Descriptive statistic of surface non-flatness of thaw-mounted brain section, spin-flattened bone section, and direct-mounted bone section. (C) Frequency distribution of points away from the center plane with a bin size of 5 µm.

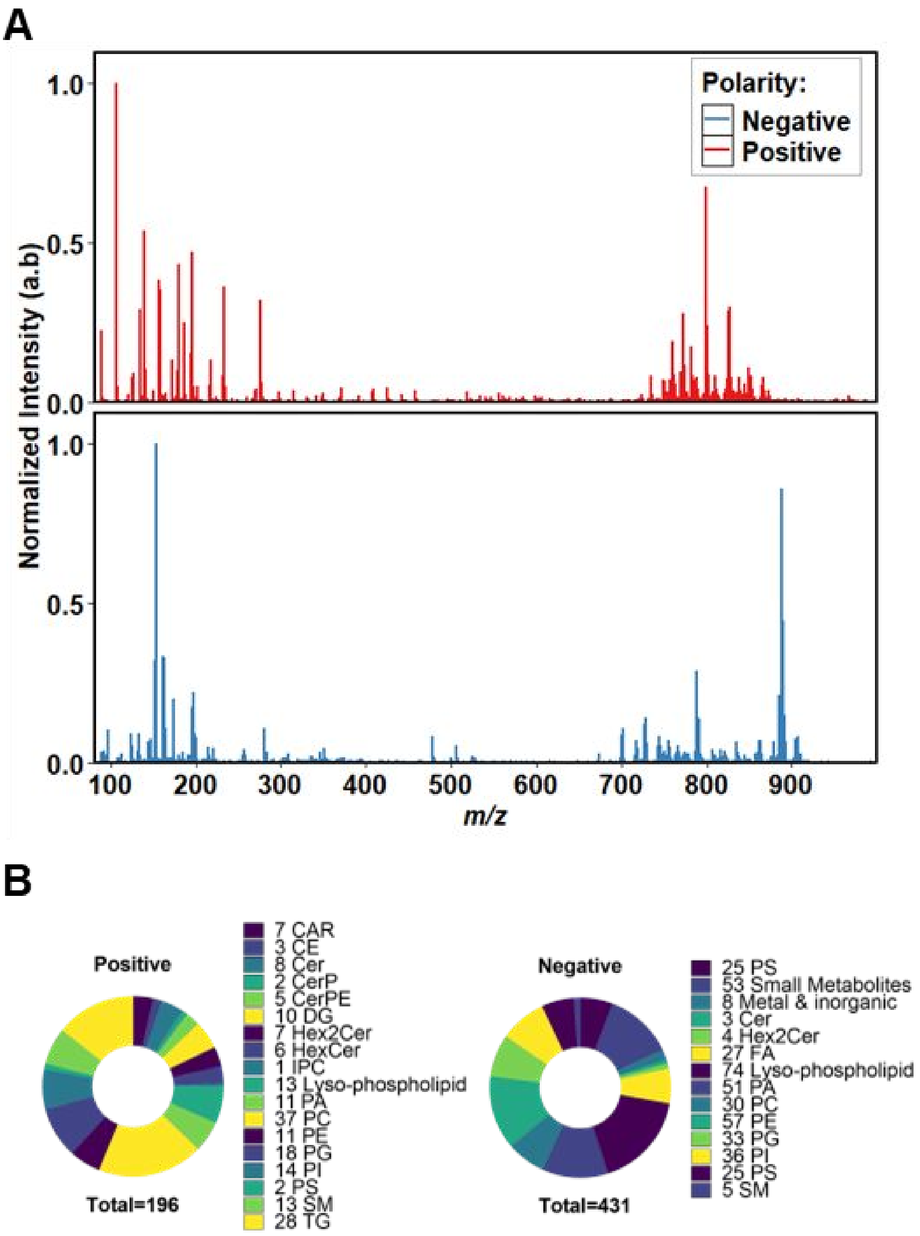


**Figure S6. Representative averaged mass spectra in both positive and negative ion mode and merged ion image of the rat tibia epiphysis.** (A) averaged mass spectra obtained in both positive (red) and negative (blue) ion modes on a MALDI-TOF instrument. Intensity was normalized to base peaks. Red and blue combs on the top and bottom x-axis represent a positive hit of metabolites at respective *m/z*. (B) number of metabolites assignments for positive and negative ion mode respectively.

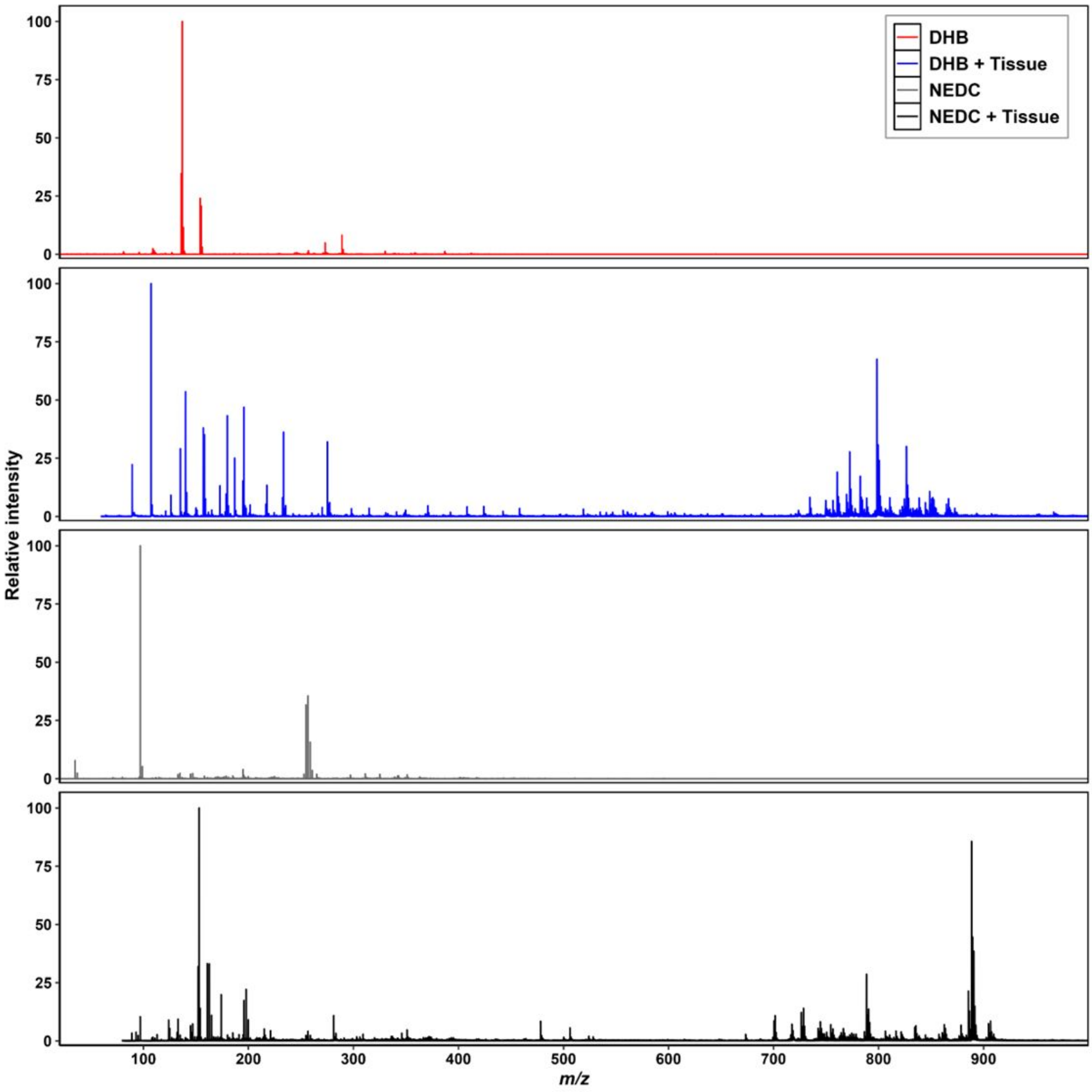


**Figure S7. Representative mass spectrum of 1,2 Dihydroxybenzoic acid (DHB), metabolome measured with DHB, (N-naphthyl) ethylenediamine hydrochloride (NEDC), and metabolome measured with NEDC.**

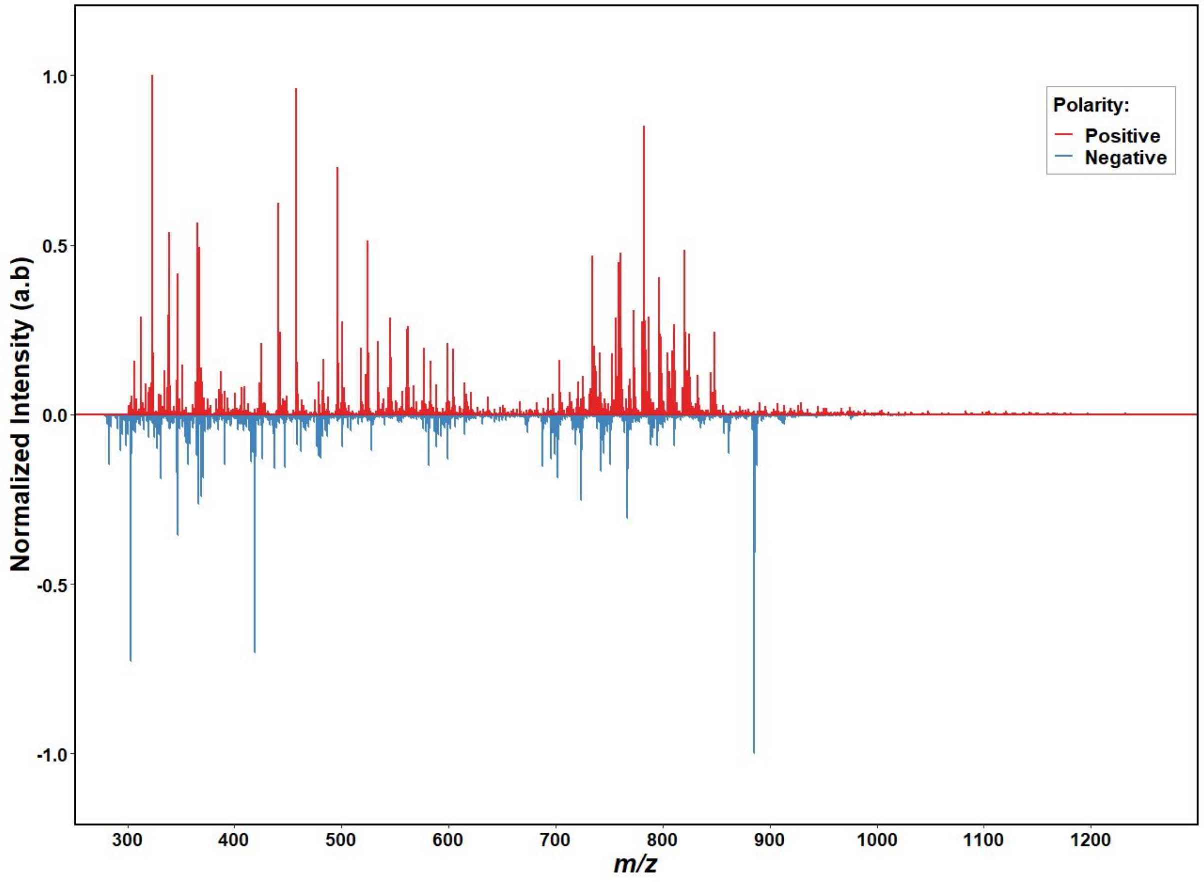


**Figure S8. Representative mass spectrum was acquired using a high mass resolution Q-TOF instrument.** Intensity was normalized to the base peak. The negative ion mode mass spectrum was colored blue, and the positive was colored red.

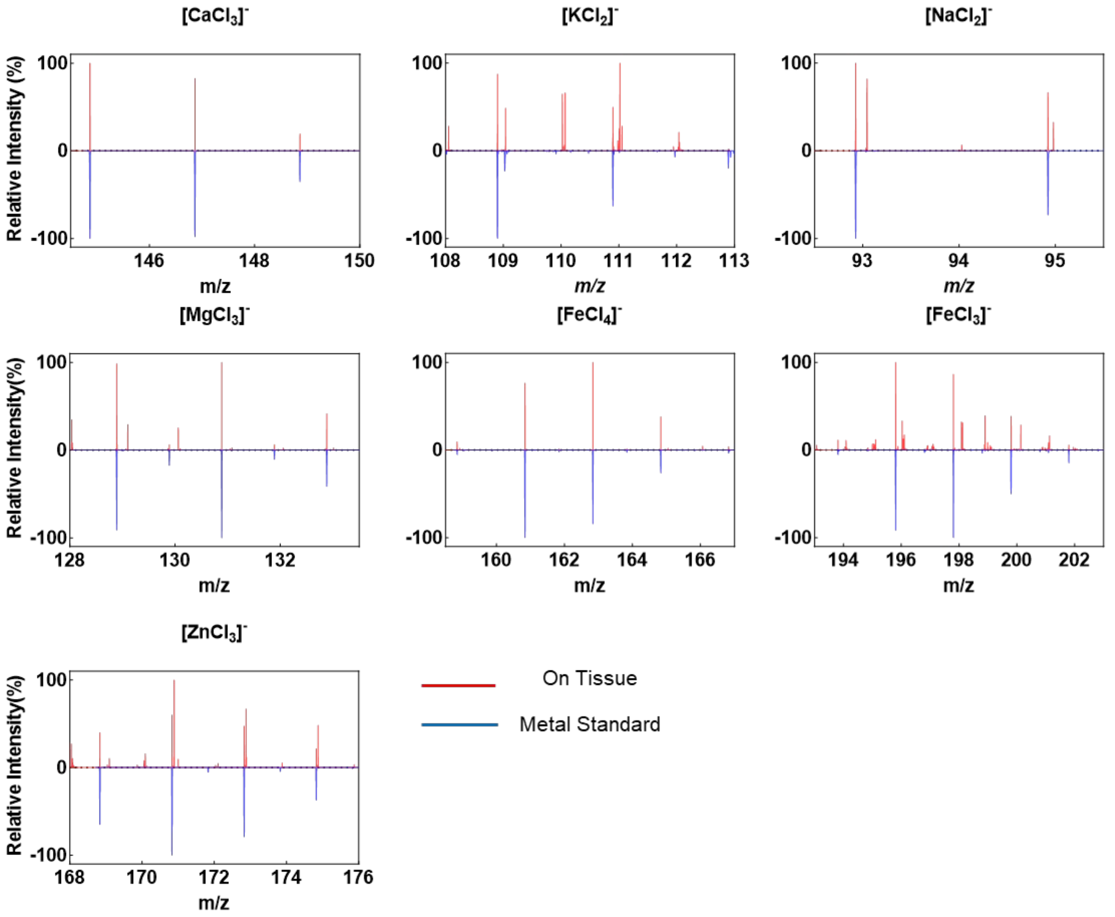


**Figure S9. High-resolution mass spectra of seven metal chloride adducts were obtained in negative mode.** Experimental values were plotted in red, and isotopic simulation plotted in blue.

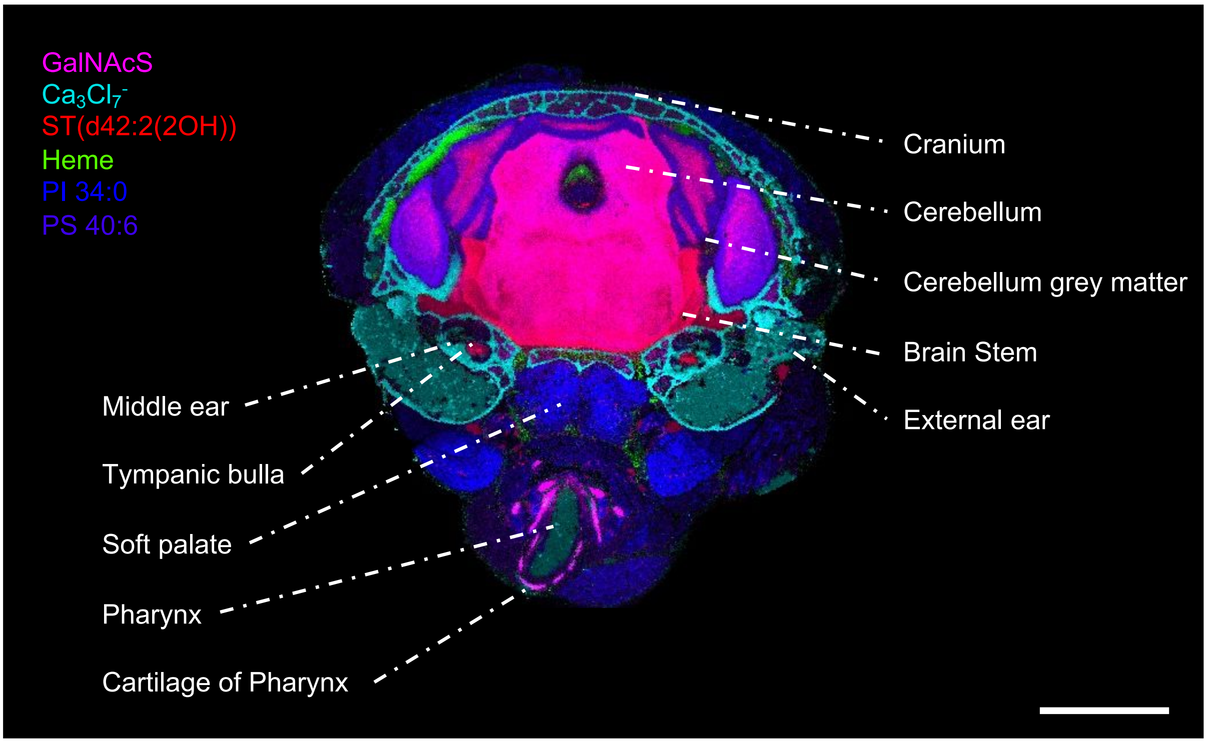


**Figure S10. Overlaid ion image of mouse cranium with anatomic annotation.**

180 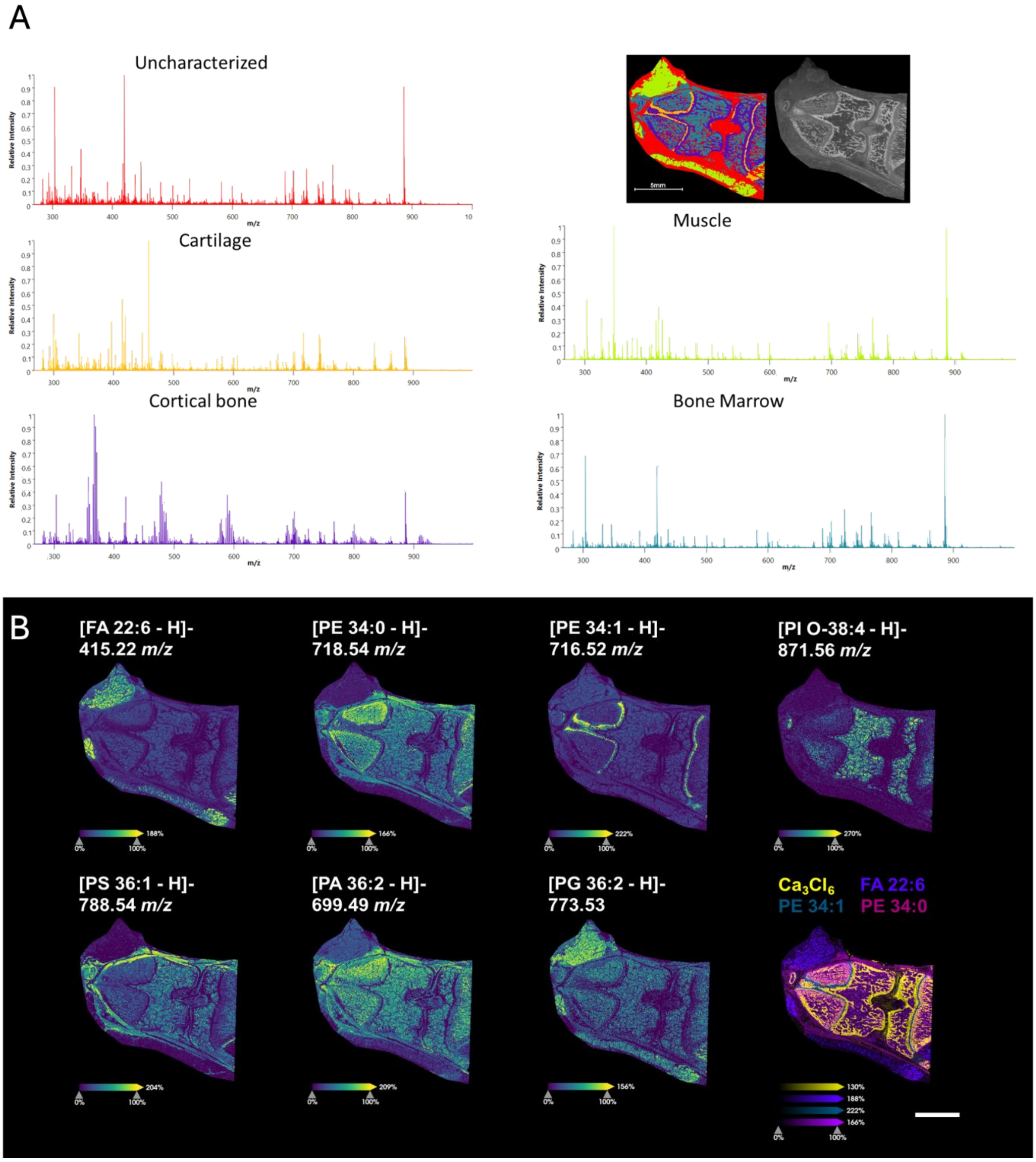


181 **Figure S11. Averaged mass spectra and 30 µm lateral resolution ion images of cartilage,**
182 **bone marrow, cortical bone, and muscle area.** (A) Ion images of FA 22:6, PE 34:0, PE 34:1,
183 PI O-38:4, PS 36:1, PA 36:2, PG 36:2, and merged ion images of $Ca_3Cl_6$, PE 34:1, FA22:6 and
184 PE 34:0. The scale bar at the lower left represents 4 mm.

185

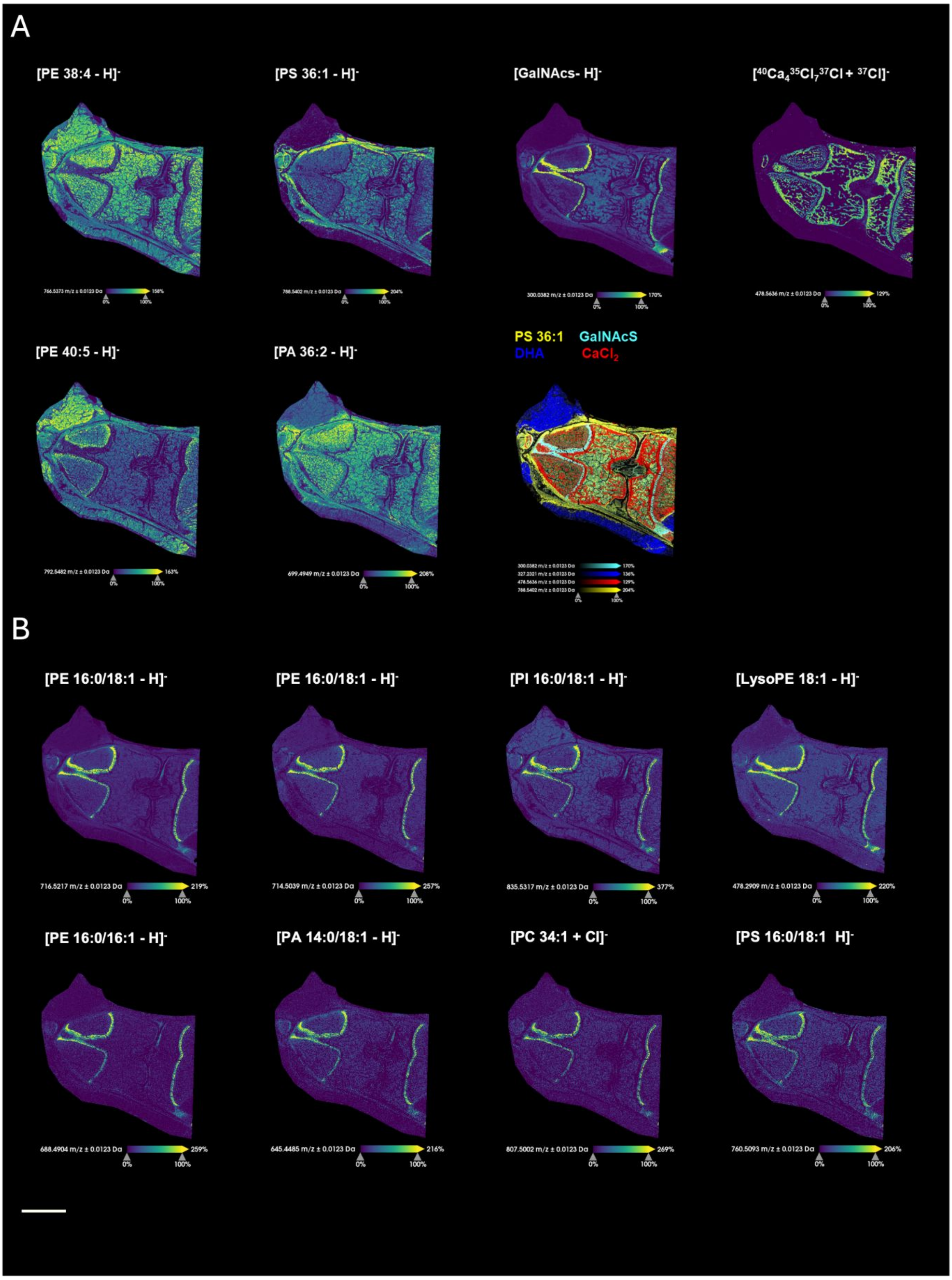


**Figure S12. Representative 30 µm lateral resolution ion images of a rat joint generated by negative ion mode MALDI-MSI.** (A) **showing spatial preference of different metabolites in muscle, cartilage, mineral bones, and bone marrow (B)** Ion images of lipid

species highly enriched in the cartilage region of rat joint (B). The scale bar at the lower left corner of Panel (B) represents 4 mm.

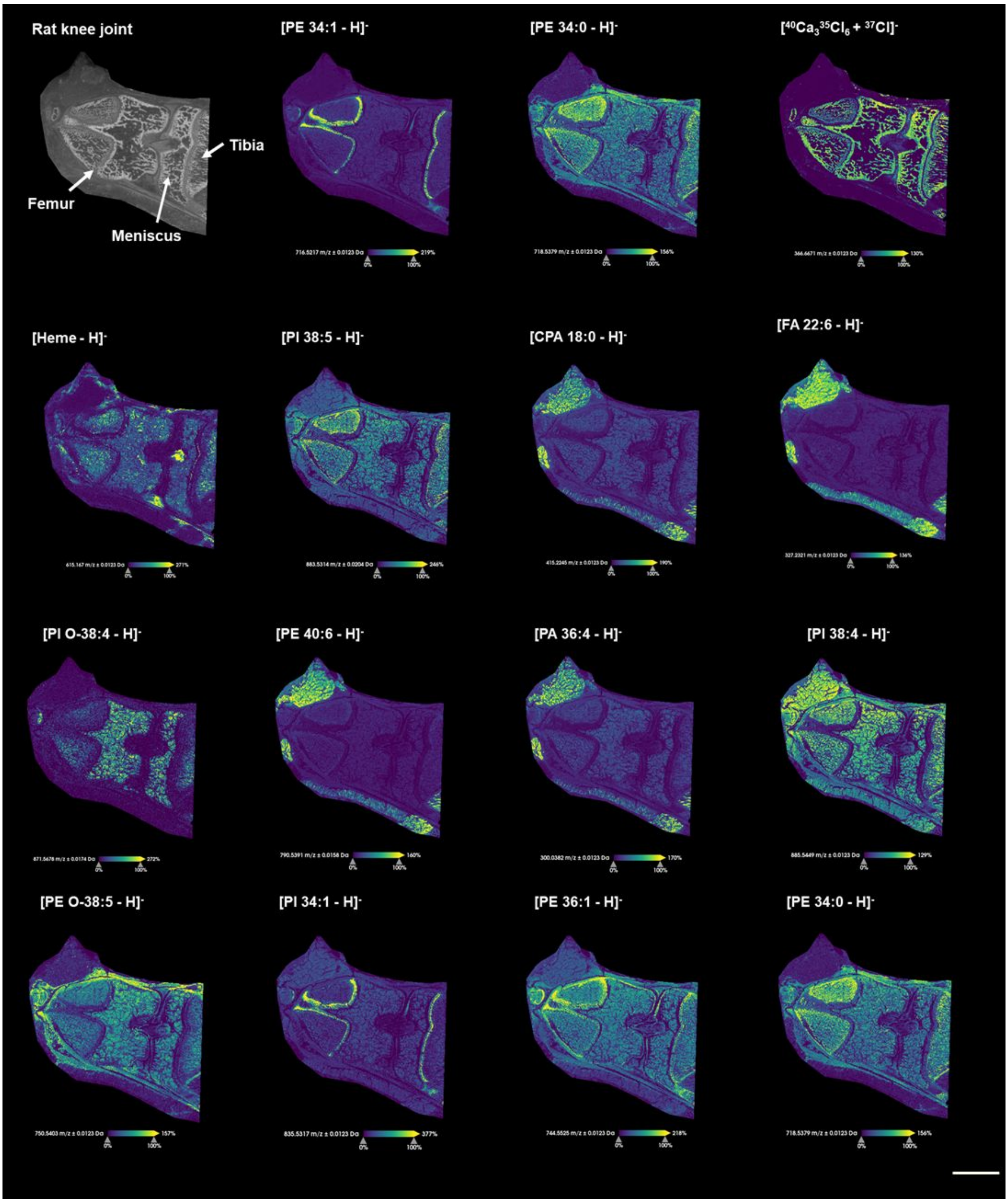


**Figure S13. Representative 30 μm lateral resolution ion images of rat joint generated by negative ion mode MALDI-MSI.** The First image is an optical scan with anatomic annotation indicating the relative position of the femur, meniscus, and tibia. The scale bar at the lower right represents 4 mm.

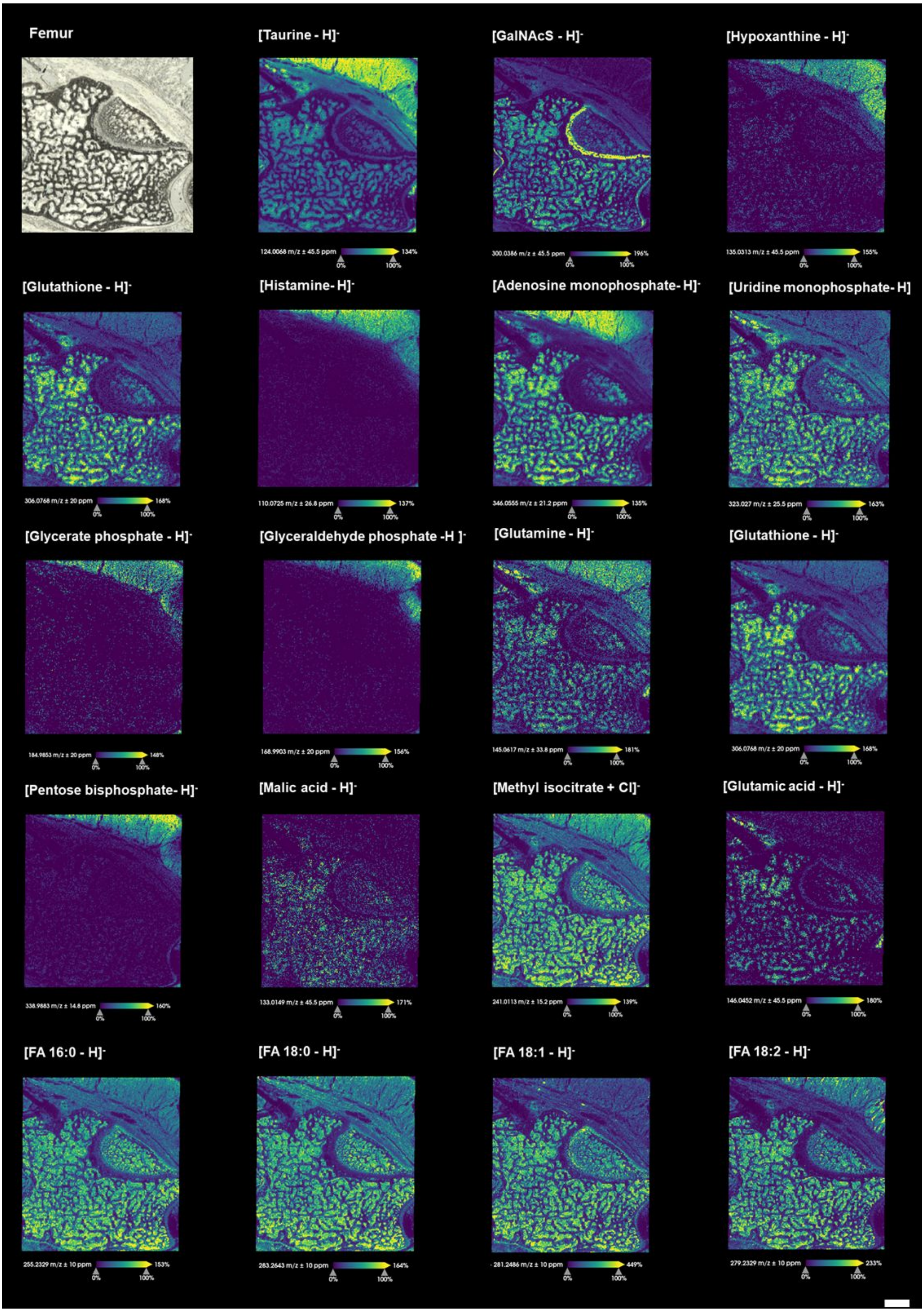
Femur
[Taurine - H]-
[GalNAcS - H]-
[Hypoxanthine - H]-
[Glutathione - H]-
[Histamine- H]-
[Adenosine monophosphate- H]-
[Uridine monophosphate- H]
[Glycerate phosphate - H]-
[Glyceraldehyde phosphate -H ]-
[Glutamine - H]-
[Glutathione - H]-
[Pentose bisphosphate- H]-
[Malic acid - H]-
[Methyl isocitrate + Cl]-
[Glutamic acid - H]-
[FA 16:0 - H]-
[FA 18:0 - H]-
[FA 18:1 - H]-
[FA 18:2 - H]-

**Figure S14. Representative 30 µm lateral resolution ion images of polar metabolites (m/z < 500) in rat femur.** An optical scan of the image of the femur area is shown in the first insert. The scale bar shown in the lower right corner represents 1 mm.

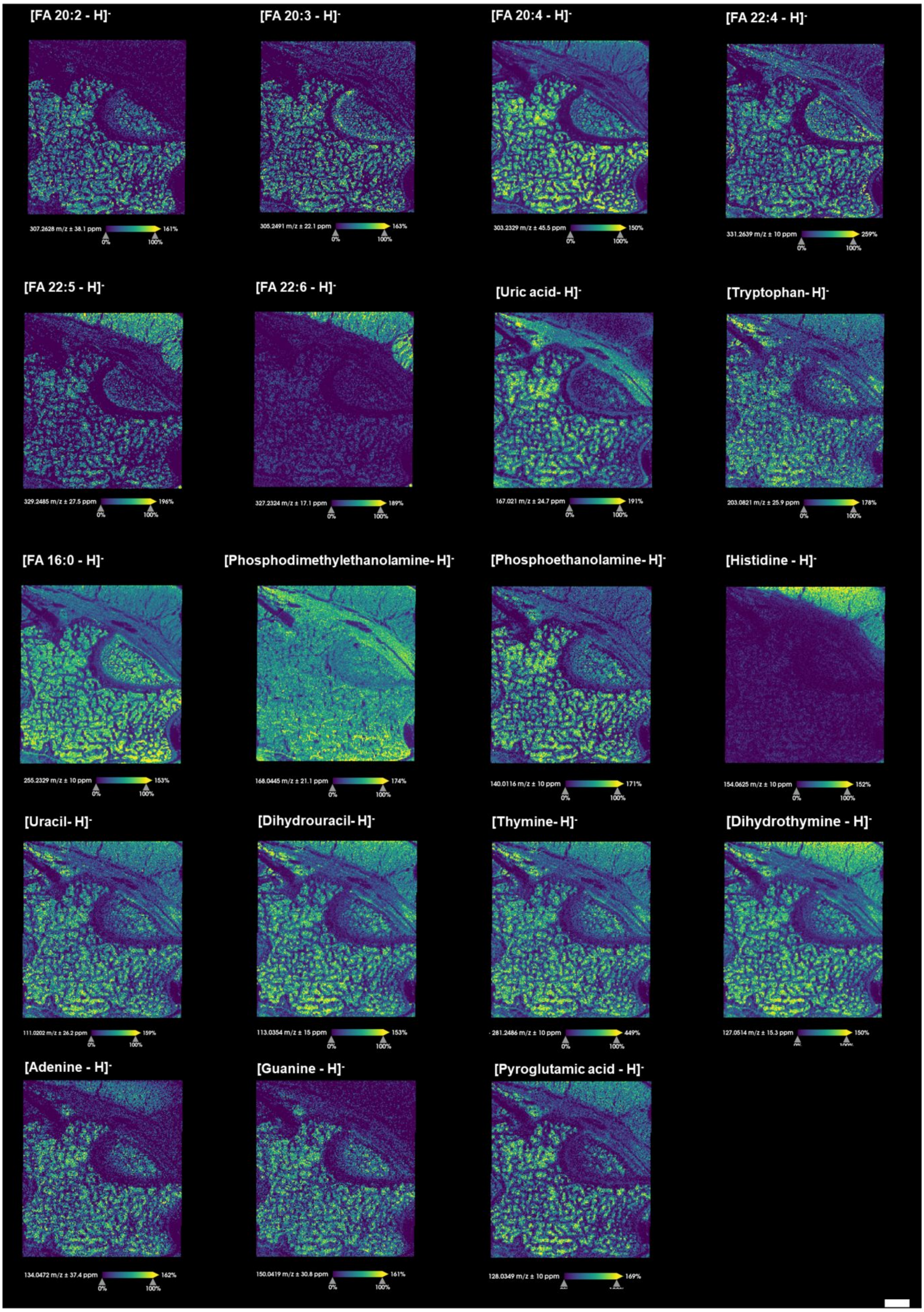

[FA 20:2 - H]-
307.2628 m/z ± 38.1 ppm 161% 0% 100%
[FA 20:3 - H]-
305.2491 m/z ± 22.1 ppm 163% 0% 100%
[FA 20:4 - H]-
303.2329 m/z ± 45.5 ppm 150% 0% 100%
[FA 22:4 - H]-
331.2639 m/z ± 10 ppm 259% 0% 100%
[FA 22:5 - H]-
329.2485 m/z ± 27.5 ppm 196% 0% 100%
[FA 22:6 - H]-
327.2324 m/z ± 17.1 ppm 189% 0% 100%
[Uric acid- H]-
167.021 m/z ± 24.7 ppm 191% 0% 100%
[Tryptophan- H]-
203.0821 m/z ± 25.9 ppm 178% 0% 100%
[FA 16:0 - H]-
255.2329 m/z ± 10 ppm 153% 0% 100%
[Phosphodimethylethanolamine- H]-
168.0445 m/z ± 21.1 ppm 174% 0% 100%
[Phosphoethanolamine- H]-
140.0116 m/z ± 10 ppm 171% 0% 100%
[Histidine - H]-
154.0625 m/z ± 10 ppm 152% 0% 100%
[Uracil- H]-
111.0202 m/z ± 26.2 ppm 159% 0% 100%
[Dihydrouracil- H]-
113.0354 m/z ± 15 ppm 153% 0% 100%
[Thymine- H]-
281.2486 m/z ± 10 ppm 449% 0% 100%
[Dihydrothymine - H]-
127.0514 m/z ± 15.3 ppm 150%
[Adenine - H]-
134.0472 m/z ± 37.4 ppm 162% 0% 100%
[Guanine - H]-
150.0419 m/z ± 30.8 ppm 161% 0% 100%
[Pyroglutamic acid - H]-
128.0349 m/z ± 10 ppm 169%

**Figure S15. Representative 30 µm lateral resolution ion images of polar metabolites (m/z < 500) in rat femur.** The scale bar shown in the lower right corner represents 1 mm.

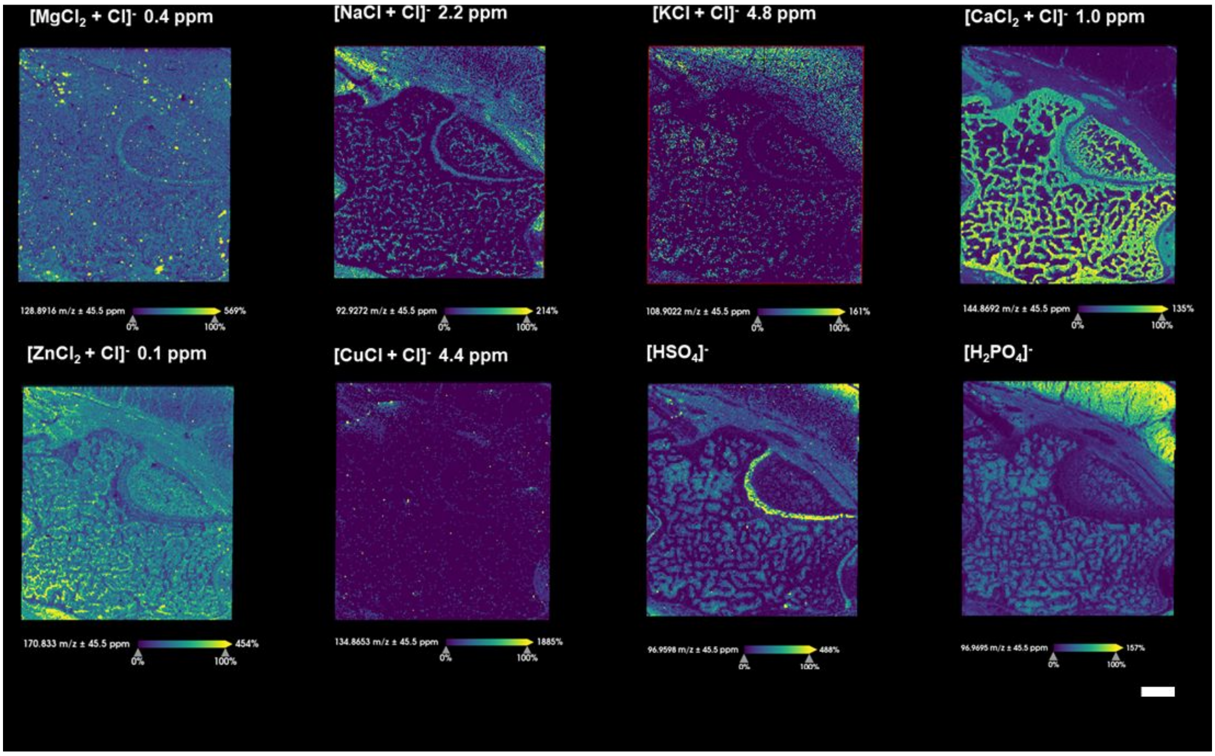


**Figure S16. Inorganic ions maps of rat femur measured at 30 µm lateral resolution.** The scale bar shown in the lower right corner represents 1 mm.

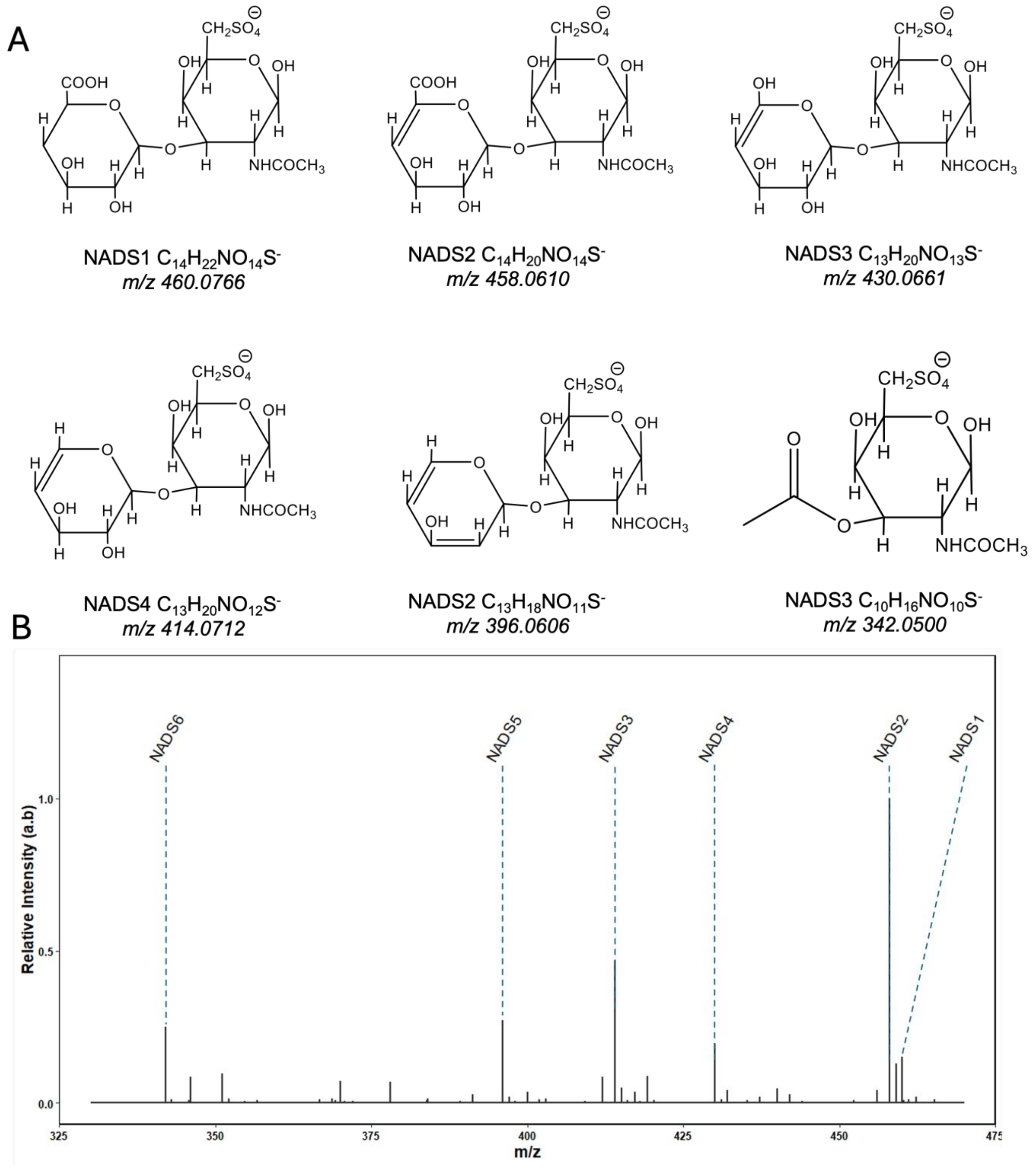


**Figure S17. The proposed structures and mass spectra of NADS1 to NADS6.** (A) mass spectra of 6 NADS. (B) Average NADS enriched region mass spectra in growth plate cartilage.

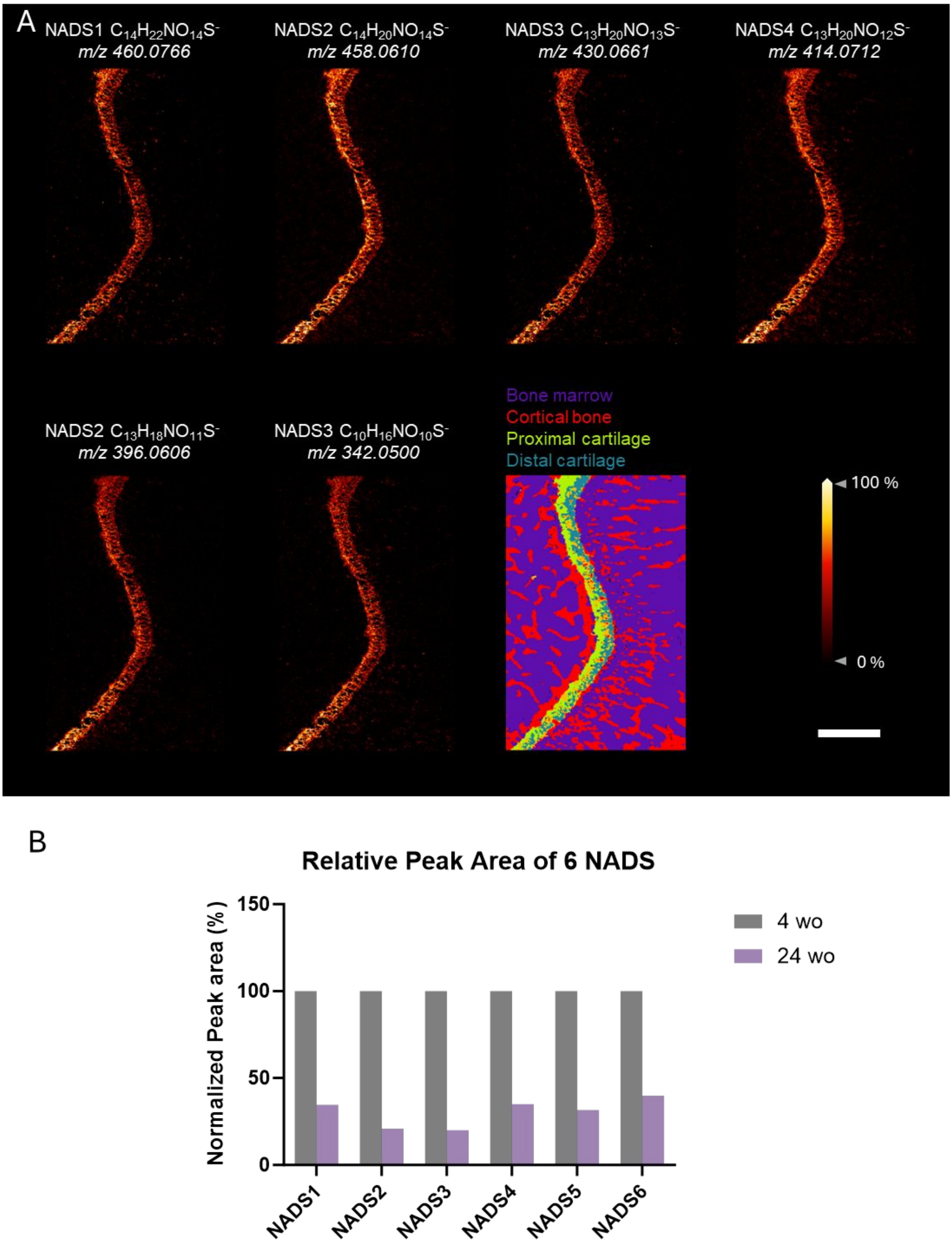


**Figure S18. Representative ion images and relative abundance of six NADSs are highly enriched in the growth plate cartilage region.** Representative ion images of six NADSs and bisecting K mean growth plate cartilage clustering map. The separated proximal and distal

cartilage clustering indicates a distinct molecular profile (A). The relative peak area of six NADSs between 4-week-old (4wo) and 24-week-old (24wo) rat growth plate cartilage. The scale bar at the lower left represents 900 μm.
